# Downregulated SPESP1‐driven fibroblast senescence decreases wound healing in aged mice

**DOI:** 10.1002/ctm2.1660

**Published:** 2024-05-19

**Authors:** Yun Zhong, Lei Zhou, Yi Guo, Fan Wang, Fanping He, Yufan Cheng, Xin Meng, Hongfu Xie, Yiya Zhang, Ji Li

**Affiliations:** ^1^ Department of Dermatology Xiangya Hospital Central South University Changsha Peoples Republic of China; ^2^ Hunan Key Laboratory of Aging Biology Xiangya Hospital Central South University Changsha Peoples Republic of China; ^3^ Department of Dermatology The Third Affiliated Hospital Sun Yat‐sen University Guangzhou Peoples Republic of China; ^4^ National Clinical Research Center for Geriatric Disorders Xiangya Hospital Central South University Changsha Hunan Peoples Republic of China

**Keywords:** cellular senescence, skin ageing, wound healing

## Abstract

**Background:**

Human dermal fibroblasts (HDFs) are essential in the processes of skin ageing and wound healing. However, the underlying mechanism of HDFs in skin healing of the elderly has not been well defined. This study aims to elucidate the mechanisms of HDFs senescence and how senescent HDFs affect wound healing in aged skin.

**Methods:**

The expression and function of sperm equatorial segment protein 1 (SPESP1) in skin ageing were evaluated via in vivo and in vitro experiments. To delve into the potential molecular mechanisms by which SPESP1 influences skin ageing, a combination of techniques was employed, including proteomics, RNA sequencing, immunoprecipitation, chromatin immunoprecipitation and liquid chromatography‐mass spectrometry analyses. Clearance of senescent cells by dasatinib plus quercetin (D+Q) was investigated to explore the role of SPESP1‐induced senescent HDFs in wound healing.

**Results:**

Here, we define the critical role of SPESP1 in ameliorating HDFs senescence and retarding the skin ageing process. Mechanistic studies demonstrate that SPESP1 directly binds to methyl‐binding protein, leading to Decorin demethylation and subsequently upregulation of its expression. Moreover, SPESP1 knockdown delays wound healing in young mice and SPESP1 overexpression induces wound healing in old mice. Notably, pharmacogenetic clearance of senescent cells by D+Q improved wound healing in SPESP1 knockdown skin.

**Conclusions:**

Taken together, these findings reveal the critical role of SPESP1 in skin ageing and wound healing, expecting to facilitate the development of anti‐ageing strategies and improve wound healing in the elderly.

## Graphical abstract

The role and mechanism of SPESP1/MECP2/DCN axis in wound healing of senescent skin

1. Downregulated SPESP1 accelerates cell senescence and skin aging.

2. SPESP1 retards the aging process by binding to methyl binding protein (MeCP2) to regulate DCN expression.

3. The accumulation of senescent cells resulting from the depletion of SPESP1 is a significant contributor to the deceleration of healing in aging individuals.

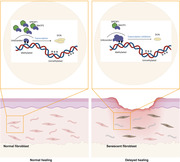

## 1 INTRODUCTION

Skin serves as the essential protective barrier for the human body by preventing dehydration, blocking ultraviolet radiation‐induced photolesions and defending against pathogens and infection.^1^, ^2^, ^3^, ^4^ In ageing individuals, the skin becomes thin and dry, leading to delayed wound healing.^1^, ^5^ Substantial evidence indicates that wounds in elderly individuals often fail to heal properly. Age‐related declines in skin healing can result in health complications and a decreased lifespan.^6^ However, the molecular mechanisms contributing to the delayed healing in aged individuals remain unclear, impeding the prospects for therapeutic advances.

Accumulated senescent cells are significant contributors to organ ageing, including skin ageing.^7^ However, the role of senescent cells in tissue regeneration is puzzling. Studies have shown that senescent cells can affect skin wound healing by secreting the senescence‐associated secretory phenotype (SASP) ^8^, ^9^ and by participating in tissue homeostasis.^10^ Conversely, senescent cells show abbreviated growth phases, enhanced resting and are delayed in response to tissue‐regenerating cues.^11^ Recently, fibroblasts, the major stromal cells of the skin, have been identified as the majority of senescent cells in aged skin.^12^, ^13^ The proliferative and collagen‐producing capacity of senescent fibroblasts is reduced, implying the age‐related decline of skin wound healing.^14^, ^15^, ^16^ Decorin (DCN) synthesized and secreted by dermal fibroblasts is thought to be an important player in maintaining skin and tendon integrity.^17^ However, how senescent fibroblasts affect skin healing in elderly individuals remains to be further explored.

Sperm equatorial segment protein 1 (SPESP1) was first cloned and characterized in humans, characterized by the involvement in sperm‐egg fusion.^18^, ^19^ Recently, SPESP1 was proposed to be an effective prognostic marker for malignant tumours, such as breast cancer.^20^ Moreover, SPESP1 is hypermethylated in congenital heart disease and is implied as a new biomarker and potential intervention target for congenital heart disease.^21^ By analysing published transcriptome data, we noted the abnormal expression of SPESP1 in multiple senescence cell models. However, its exact roles and mechanisms in ageing and tissue regeneration/wound healing remain unknown.

Here, we define the critical role of SPESP1 in ameliorating fibroblast senescence, protecting against skin ageing and improving wound healing in elderly people. Mechanistically, SPESP1 directly targets methyl‐binding protein (MeCP2), resulting in the upregulation of DCN expression to ameliorate cell senescence. This process, in turn, influences skin homeostasis and regeneration. This study demonstrates the protective role of SPESP1 against skin ageing, offering potential implications for advancing anti‐ ageing strategies and improving skin healing in the elderly.

## 2 RESULTS

### 2.1 SPESP1 was downregulated in aged skin tissues and senescent human dermal fibroblasts

**FIGURE 1:**
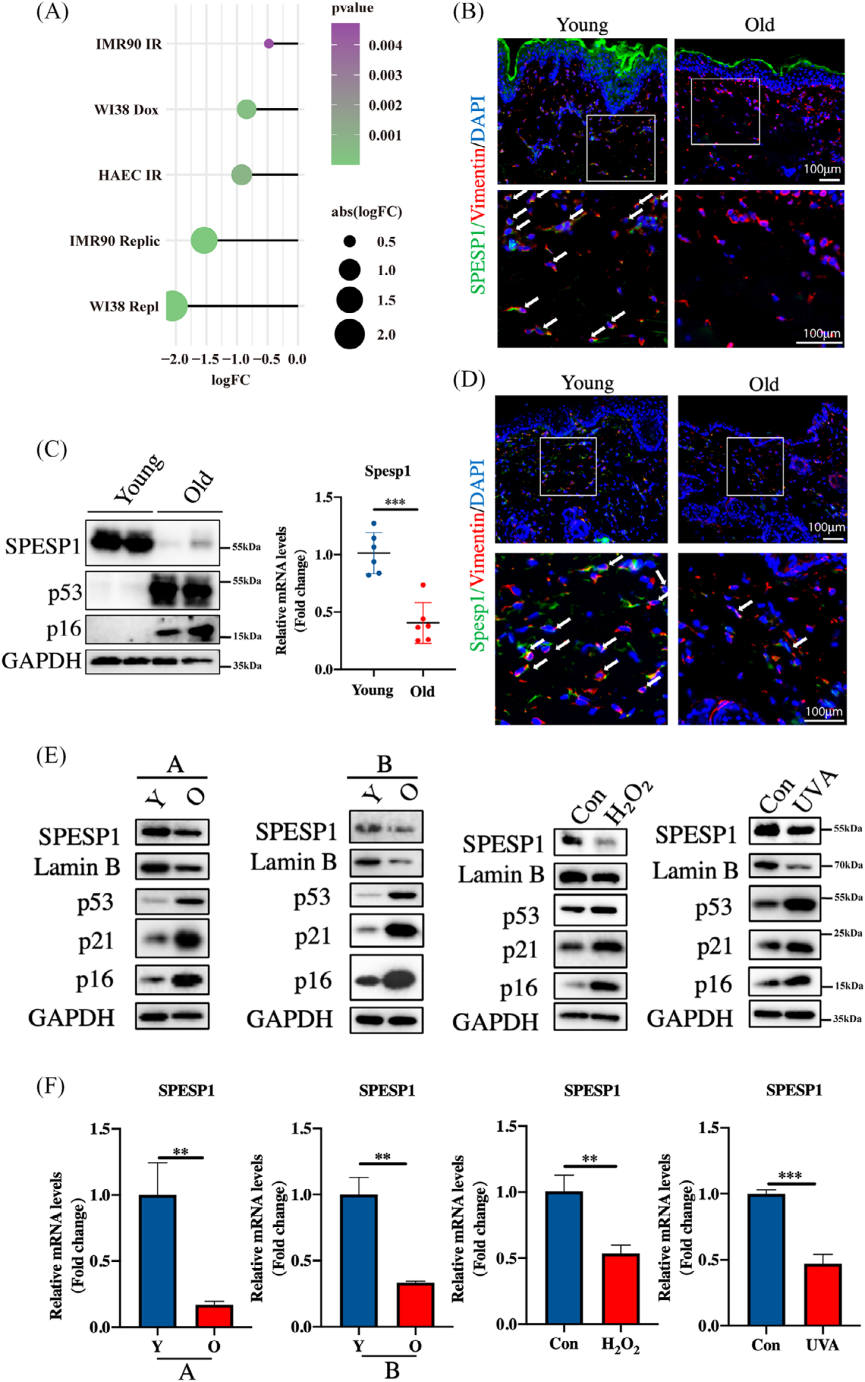
The levels of SPESP1 are low in the aged skin tissue and HDFs. (A) The SPESP1 expression in IR/Dox/Replic‐induced senescent IMR90 (human embryo lung fibroblasts), WI38 (human embryonic lung fibroblasts), HACE (human aortic endothelial cells) and HUVEC (human umbilical vein endothelial cells) in GSE130727 dataset. (B) Immunofluorescence (IF) staining of SPESP1 and Vimentin in human skin of young (*n* = 8, mean age 23.75) and old groups (*n* = 6, mean age 76.5). (C) qPCR (right) and western blotting (left) showed the expression levels of SPESP1 in young (*n* = 8, mean age 2 months) and old (*n* = 8, mean age 20 months) mice skin. (D) IF staining of SPESP1 and Vimentin in young (*n* = 8, mean age 2 months) and old mice skin (*n* = 8, mean age 20 months). (E) and (F) HDFs were used to construct passage senescence (A, B: two different individuals; Y: young passage; O: old passage), UVA‐induced senescence, and H_2_O_2_‐induced senescence. After verifying the successful model construction, the expression of SPESP1 in senescent HDFs was analysed by RT‐qPCR and Western blotting. The data are shown as the mean ± SEM; ∗*p* < 0.05; ∗∗*p* < 0.01; ∗∗∗*p* < 0.001; ∗∗∗∗*p* < 0.0001; ns, not significant.

Firstly, we screened senescence‐related genes in nine models of senescent cells using the GSE130727 dataset. As shown in Figure 1A, SPESP1 was downregulated in IR‐induced, Dox‐induced and replicative senescence cells. Next, the SPESP1 expression in skin tissues was further analysed using 2‐ and 20‐month‐old C57BL/6J mice. The protein and mRNA levels of SPESP1 were significantly downregulated in the skin tissue of aged mice (Figure 1C and Supplementary Figure 1A). Immunofluorescent staining revealed that SPESP1 was mainly expressed in human dermal fibroblasts (HDFs) (Figure 1D). Similar results were subsequently observed in human skin tissues of young and old healthy individuals from the Xiangya Hospital of Central South University (Figure 1B). Next, we explored the levels of SPESP1 in different HDFs senescence models. We constructed replicative senescence, Ultraviolet Radiation A (UVA)‐induced senescence and H_2_O_2_‐induced senescence models, respectively, and detected the p16, p21, p53 and Lamin B expression in these models. The expression of p16, p21 and p53 was elevated, and Lamin B was decreased, indicating that the senescence models were successfully constructed (Figure 1E). Consistent with the expression level in vivo, the mRNA and protein levels of SPESP1 were evidently downregulated in multiple senescent cells (Figure 1E,F and Supplementary Figure 1B). Thus, these findings suggest that SPESP1 is lowly expressed in human ageing skin tissues and senescent HDFs.

### 2.2 SPESP1 inhibits cellular senescence and delays skin ageing

**FIGURE 2:**
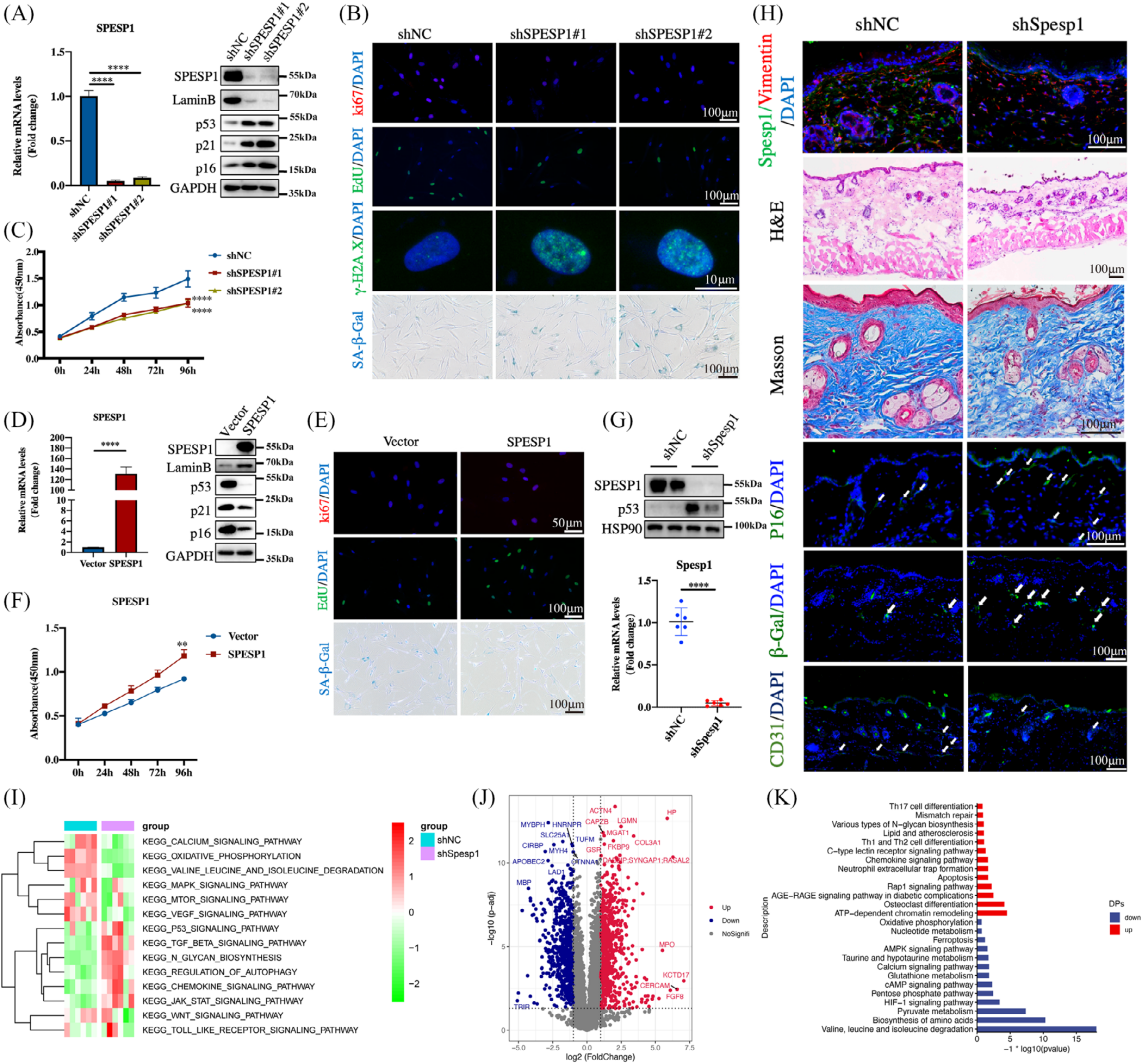
Knockdown of SPESP1‐induced cellular senescence and skin ageing. Young‐passages HDFs (PD  <  10) were infected with shSPESP1 or negative control shNC. (A) The shSPESP1 downregulated the mRNA expression of SPESP1 by qRT‐PCR (right) and the protein levels of SPESP1, p53, p21, p16 and LaminB by western blotting (left). (B) Immunofluorescence staining of Ki67, Edu and γ‐H2A.X, SA‐β‐Gal staining in HDFs upon shRNA‐mediated knockdown of SPESP1. (C) Cell proliferation measured by CCK8. Senescent HDFs (PD   > 35) were transfected with SPESP1 or Vector. (D) The SPESP1 upregulated the mRNA expression of SPESP1 (right). The protein levels of SPESP1, p53, p21, p16 and LaminB by Western blotting (left). (E) Immunofluorescence staining of Ki67, Edu and SA‐β‐Gal staining in HDFs upon overexpression of SPESP1. (F) Cell proliferation measured by CCK8. 6‐week‐old C57BL/6J mice dorsal skin were injected with 10 µL high‐titre lentivirus containing shSPESP1 (shSPESP1, > 5 × 10^8^ cfu/mL) or shNC once every other day for 8 weeks, and sacrificed. (G) The protein (up) and mRNA expression (down) in shSpesp1 skin of mice (*n* = 5 mice per group, mean age 2 months). (H) H&E staining, Masson staining and immunofluorescence staining of SPESP1/Vimentin, p16, CD31, β‐Gal in skin tissues. (I) The heatmap of GSVA analysis from skin proteome. (J) The volcano plot revealed the DEPs between the control and Spesp1sh groups. (K) The KEGG enrichment analysis of DEPs. The data are shown as the mean ± SEM; ∗*p* < 0.05; ∗∗*p* < 0.01; ∗∗∗*p* < 0.001; ∗∗∗∗*p* < 0.0001; ns, not significant.

To assess whether SPESP1 is involved in the ageing process, we knocked down or overexpressed SPESP1 in HDFs using lentivirus. The SPESP1 expression was silenced by shSPESP1 lentivirus in young HDFs (passages < 15) (Figure 2A). After knocking down SPESP1, HDFs exhibited numerous distinct signs of senescence. These included the classic senescent morphology characterized by a flattened and enlarged cellular structure, a rise in the protein levels of p16, p21 and p53, a reduction in Lamin B expression and an enhancement in senescence‐associated β‐galactosidase (SA‐β‐Gal) staining, as depicted in Figure 2A, B and Supplementary Figure 2A,C. Moreover, CCK8, Ki67 immunofluorescence, Edu incorporation and flow cytometry cell cycle results indicated that knockdown of SPESP1 repressed growth and induced G0/G1 phase arrest of HDFs, and no evidence of apoptosis was measured in these cells (Figure 2B,C and Supplementary Figure 2B,D). Furthermore, SPESP1 knockdown resulted in increased expression of γH2A.X and SASP (Figure 2B and Supplementary Figure 2E).

To determine the role of SPESP1 during passage senescence, rescue experiments were performed by overexpressing SPESP1 in passage‐senescent HDFs (passages > 35) (Figure 2D and Supplementary Figure 2F). As expected, overexpression of SPESP1 alone significantly delayed the ageing phenotype in HDFs with reduced SA‐β‐gal positive cells (Figure 2D,E and Supplementary Figure 2H). CCK8, Ki67 immunofluorescence, Edu incorporation and flow cytometry cell cycle experiments further supported the ability of SPESP1 to maintain proliferation after overexpression (Figure 2E,F and Supplementary Figure 2G). In addition, overexpression of SPESP1 also reduced SASP levels (Supplementary Figure 3A).

To identify the role of SPESP1 in skin ageing, we used the intradermal injection of lentiviruses expressing short hairpin RNA (shRNA), which results in efficient knockdown of SPESP1 in the skin. Consistent with the findings in vitro, the SPESP1 knockdown led to significant thinning of the dermis by HE staining, and SPESP1 knockdown also reduced and loosely arranged collagen fibres by Masson staining (Figure 2H), and induced a significant senescence‐related phenotype with high p53 and p21 levels (Figure 2G  and Supplementary Figure 3B,D). Immunofluorescence analysis revealed a significant increase in cells positive for p16 and β‐Gal staining (Figure 2H and Supplementary Figure 3C), indicating the increased senescent cells in SPESP1 knockdown skin tissue. The SPESP1‐silenced skin showed a notable elevation of SASP, including Tgfbeta1, Il‐1b and Mmp9. Conversely, genes related to the cell cycle (Ccnd1) and collagen fibril synthesis (Col1a1) showed a marked decrease in expression, as detailed in Supplementary Figure 1F. Interestingly, we also found that angiogenesis was significantly reduced in the shSpesp1‐treated skin compared with shNC‐treated skin in mice (Figure 2H and Supplementary Figure 3C).

**FIGURE 3:**
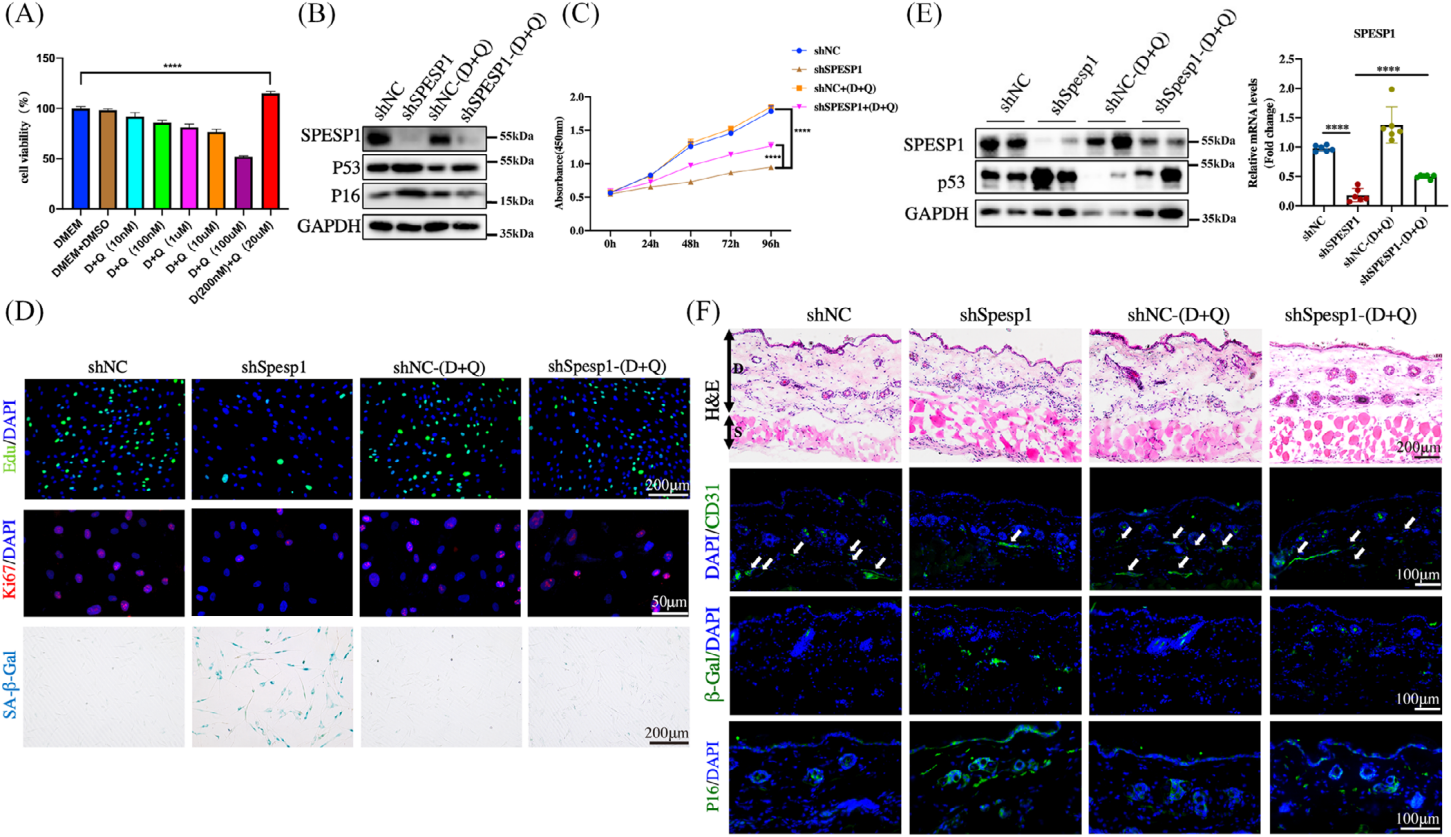
Senolytic drug delays shSPESP1‐induced skin ageing by removing senescent cells. (A) Detection of different concentrations of D+Q drugs (10 nM, 100 nM, 1 µM, 10 µM, 100 µM, 200 nM + 20 µM) on the cell viability of HDF. (B) The expression levels of SPESP1, p16 and p53 were detected by Western blotting; GAPDH: internal reference. (C) Cell proliferation measured by CCK8. (D) Effects of D+Q on cell cycle and senescence of HDF in shNC and shSPESP1 cells demonstrated by immunofluorescence. (E) The expression levels of SPESP1 and p53 were detected by Western blotting (left) and qRT‐PCR (right) in shSpesp1 mice treated with D+Q. GAPDH: internal reference. (F) D+Q treatment increased skin thickness as assessed by HE staining. Treatment with D+Q reduced p16 and β‐Gal positive cells and increased CD31 expression in skin tissue as assessed by immunofluorescence staining. *n* = 6 mice per group, mean age 2 months. The data are shown as the mean ± SEM; ∗*p* < 0.05; ∗∗*p* < 0.01; ∗∗∗*p* < 0.001; ∗∗∗∗*p* < 0.0001; ns, not significant.

Next, the control and shSpesp1 skin (*n* = 6) were collected for mass spectrometry proteomics to identify potential signalling pathways linking SPESP1 knockdown‐mediated skin ageing. The principal component analysis revealed the distinct separation of control and SPESP1‐knockdown skin tissues (Supplementary Figure 3E). GSVA (gene set variation analysis) showed that ageing‐related biological processes were significantly dysregulated in shSpesp1 skin compared with control skin (Figure 2I) including the p53 pathway, and inflammatory pathways were upregulated. In contrast, the oxidative stress, amino acid degradation, mitogen‐activated protein kinases (MAPK), mammalian target of rapamycin (mTOR) and vascular endothelial growth factor (VEGF) pathways were downregulated in shSpesp1 skin. Next, differential expression analysis identified 1336 differentially expressed proteins (DEPs) with |log2FC| > 1 and *p* < 0.05 (Figure 2J and Supplementary Figure 3F). The enrichment analysis showed that the upregulated proteins are related to the advanced glycation end products‐receptor for advanced glycation end products (AGE‐RAGE) pathway, apoptosis and inflammatory pathways, while the downregulated proteins are related to amino acid degradation, HIF‐1 signalling pathway, cAMP signalling pathway and AMPK signalling pathway (Figure 2K). Collectively, these findings suggest that SPESP1 knockdown promoted fibroblast senescence and accelerated skin ageing in mice.

### 2.3 Senolytic drugs clear senescent fibroblasts and delay SPESP1 knockdown‐induced skin ageing

To determine the role of SPESP1 knockdown‐induced senescence cells in skin ageing, we used dasatinib and quercetin (D+Q) to clear senescent cells in vivo and in vitro.^22^, ^23^ Firstly, we assessed HDF viability across various D+Q concentrations, selecting D (200 nM) + Q (20 µM) for all subsequent cellular studies (Figure 3A). We found that D+Q treatment significantly repressed shSPESP1‐induced expression of senescence marker genes (Figure 3B and Supplementary Figure 4A). Moreover, D+Q administration boosted cell proliferation and lessened the count of SA‐β‐Gal‐positive cells in shSPESP1‐treated skin (Figure 3C, D). Concurrently, we gave oral administration of D+Q drugs to mice with SPESP1 knockdown in the skin. After D+Q treatment, the expression levels of ageing‐related markers (p16 and p53) were significantly decreased, skin thickness was increased, angiogenesis‐related genes were increased and β‐Gal staining was decreased in SPESP1 knockdown skin in mice (Figure 3E,F and Supplementary Figure 4C,D). Correspondingly, the expression of inflammation‐related genes, as well as genes associated with the cell cycle and collagen fibre production was rescued by D+Q treatment shSPESP1 HDFs and mice (Supplementary Figure 4B, D). These findings suggest that the SPESP1 knockdown induced HDF senescence, subsequently leading to skin ageing.

### 2.4 SPESP1 regulates cellular senescence by downregulating DCN

**FIGURE 4:**
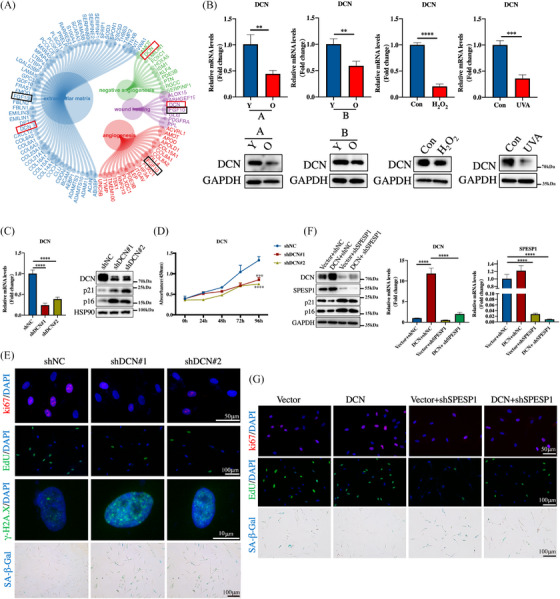
DCN is a potential target of SPESP1 and is involved in SPESP1‐mediated HDFs senescence. (A) The enrichment analysis of DEGs shows the differentially expressed genes identified between the shNC and shSPESP1 cells. (B) The expression of DCN in aged HDFs was analysed by RT‐qPCR (up) and Western blotting (down). Young‐passages HDFs (PD  <  10) were infected with shDCN or negative control shNC. (C) The shDCN downregulated the mRNA expression of DCN (left) and the protein levels of DCN, p21and p16 by Western blotting (right). (D) Cell proliferation measured by CCK8. (E) Immunofluorescence staining of Ki67, Edu and γ‐H2A.X, SA‐β‐Gal staining in HDFs upon shRNA‐mediated knockdown of DCN. A rescue assay was used to assess the reversed effect of DCN in shSPESP1‐mediated senescence. (F) The expression of SPESP1, DCN, p16 and p21 was verified at the protein level (left) and mRNA level (right). (G) Immunofluorescence staining of Ki67, Edu and SA‐β‐Gal staining indicated that DCN overexpressed could reverse shSPESP1‐induced HDFs senescence. The data are shown as the mean ± SEM; ∗*p* < 0.05; ∗∗*p* < 0.01; ∗∗∗*p* < 0.001; ∗∗∗∗*p* < 0.0001; ns, not significant.

To identify the potential downstream pathways regulated by SPESP1 in the progression of cellular senescence, RNA‐seq was performed on HDFs with SPESP1 knockdown. Transcriptome analysis identified 1038 downregulated DEGs and 705 upregulated DEGs in the shSPESP1 group compared to the shNC group with |log2FC| > 1 and adj. *p* < 0.05 (Supplementary Figure 5A, B). Gene Ontology (GO)  analysis revealed the enrichment of differentially expressed genes (DEGs) in the extracellular matrix, angiogenesis and wound healing‐related pathways. Among these DEGs, DCN and FGF10 were involved in these pathways (Figure 4A). Consistent with this result, the proteomic analysis of primary skin fibroblasts showed that the wound healing pathway and DCN, but not FGF10, were negatively correlated with p16, an important marker of ageing (Supplementary Figure 5C). DCN is a small extracellular matrix chondroitin/dermatan sulfate proteoglycan, it is involved in extracellular matrix assembly and plays a critical role in cutaneous wound healing.^24^ Therefore, DCN was selected for further analysis. It was that DCN was downregulated in multiple senescent cell models (Supplementary Figure 5D). Additionally, both the protein and mRNA levels of DCN were significantly reduced in senescent cells and could be regulated by SPESP1, which was the same as the results of the transcriptome (Figure 4B and Supplementary Figure 5E,F). Since DCN has potent anti‐inflammatory, cytokine inhibitory and anti‐fibrogenic effects, all these data prompted us to investigate whether SPESP1 delays cellular senescence by activating DCN.^25^

We then examined the effect of DCN on cellular senescence by knocking down/overexpressing DCN in HDFs. DCN knockdown/overexpression significantly increased/decreased the expression of senescence effectors P16, P21 and P53 (Figure 4C and Supplementary Figures 5G and 6A), induced/repressed cell cycle arrest in the G1 phase (Figure 4D,E and Supplementary Figures 5H and 6B–D). The SASP expression was also inhibited/promoted by DCN knockdown/overexpression (Supplementary Figures 5I and 6E).

To further examine whether DCN involved SPESP1‐knockdown‐accelerated HDFs senescence, we overexpressed DCN in SPESP1‐depleted cells. Overexpressed DCN rescued senescence phenotypes, including decreased expression of senescence‐associated molecules p16 and p21 (Figure 4F), induced cell proliferation, Edu incorporation and increased Ki67 immunoreactivity (Figure 4G and Supplementary Figure 6F). Consistently, the increase of SA‐β‐gal staining and SASP by silencing of SPESP1 was significantly reversed by overexpressed DCN (Figure 4G and Supplementary Figure 6G,H). All these data suggest that SPESP1 attenuates cellular senescence by targeting DCN.

### 2.5 SPESP1 interacts with MeCP2 to regulate DCN hypermethylation and repress its transcription

**FIGURE 5:**
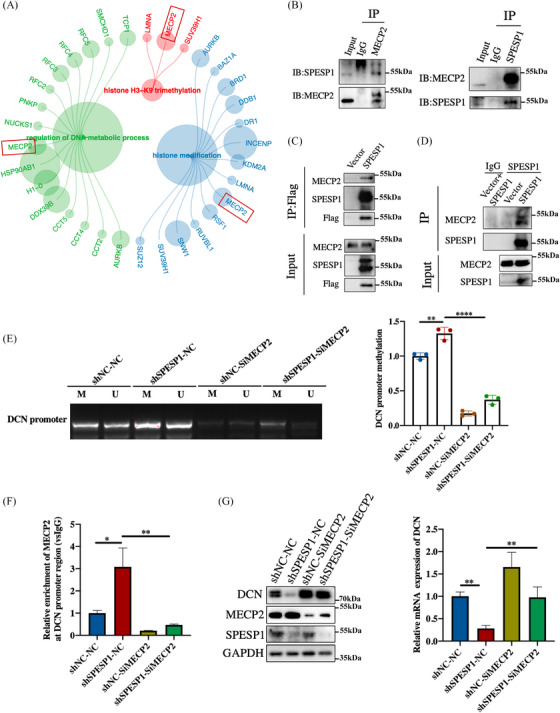
SPESP1 binds to MeCP2 to methylate the promoter region of DCN and repress its expression. (A) Mass spectrometry analysis of SPESP1‐interaction proteins. SPESP1 interactions with MeCP2 were confirmed by endogenous co‐immunoprecipitation (B) and semi‐endogenous co‐immunoprecipitation (C, D). (E) Methylation status of the DCN promoter region after knockdown of MeCP2 in SPESP1 knockdown HDFs. (U: unmethylation; M: methylation). (F) Enrichment of MeCP2 in the DCN promoter region characterized by ChIP experiments. (G) The mRNA (right) and protein expression (left) of DCN in HDFs with MeCP2 knockdown. The data are shown as the mean ± SEM; ∗*p* < 0.05; ∗∗*p* < 0.01; ∗∗∗*p* < 0.001; ∗∗∗∗*p* < 0.0001; ns, not significant.

To elucidate the mechanism through which SPESP1 influences DCN transcription, we investigated SPESP1‐interacting proteins using IP combined with mass spectrometry. The combined use of immunoprecipitated and liquid chromatography‐mass spectrometry (LC‐MS) analysis identified MeCP2 as an interacting protein of SPESP1 (Figure 5A). This interaction was further validated through the co‐immunoprecipitation of endogenous SPESP1 and MeCP (Figure 5B) and by co‐precipitation of Flag‐SPESP1 with endogenous MeCP2 (Figure 5C). Moreover, overexpressed SPESP1 significantly enhanced the interaction with MeCP2 (Figure 5D).

Methyl‐CpG‐binding protein 2 (MeCP2) can bind to cytosine methylation sites in the genome to affect gene transcription with a well‐known methyl‐CpG‐binding domain (MBD).^26^, ^27^, ^28^ According to the protein structure, SPESP1 was divided into two segments and MeCP2 was divided into three segments to verify the specific binding region of SPESP1 and MeCP2. Interestingly, we found that the MBD region of MECP2 mainly binds to the S1 fragment of SPESP1 (Supplementary Figure 7A,B). Therefore, we speculate that SPESP1 binds to the MBD region of MeCP2 to decrease its catalytic activity, which subsequently leads to hypomethylation of the DCN promoter and subsequent transcriptional activation of DCN. To verify this conjecture, we subsequently examined the methylation status of the DCN promoter region after silencing SPESP1 and MeCP2 in HDFs using methylation‐specific polymerase chain reaction (MSP) experiments. Methylation levels of the DCN promoter region significantly increased after SPESP1 knockdown. Conversely, treatment with si‐MeCP2 resulted in a rise in methylation levels. (Figure 5E and Supplementary Figure 7C). Chromatin immunoprecipitation (ChIP) assays suggested that MeCP2 enriched in the DCN promoter region was significantly increased after SPESP1 knockdown, which was rescued by si‐MeCP2 treatment (Figure 5F). We also found that mRNA and protein levels of DCN were decreased after SPESP1 knockdown, which was rescued by si‐MeCP2 treatment (Figure 5G). Moreover, H3K27me3 levels markedly increased in the shSPESP1 treatment group, whereas the contrary finding was observed by si‐MeCP2 treatment (Supplementary Figure 7D). Taken together, the obtained data suggest that SPESP1 induced DCN transcription by interacting with MeCP2 to decrease its catalytic activity.

### 2.6 SPESP1 knockdown delays wound healing in young mice and SPESP1 overexpression induces wound healing in old mice

**FIGURE 6:**
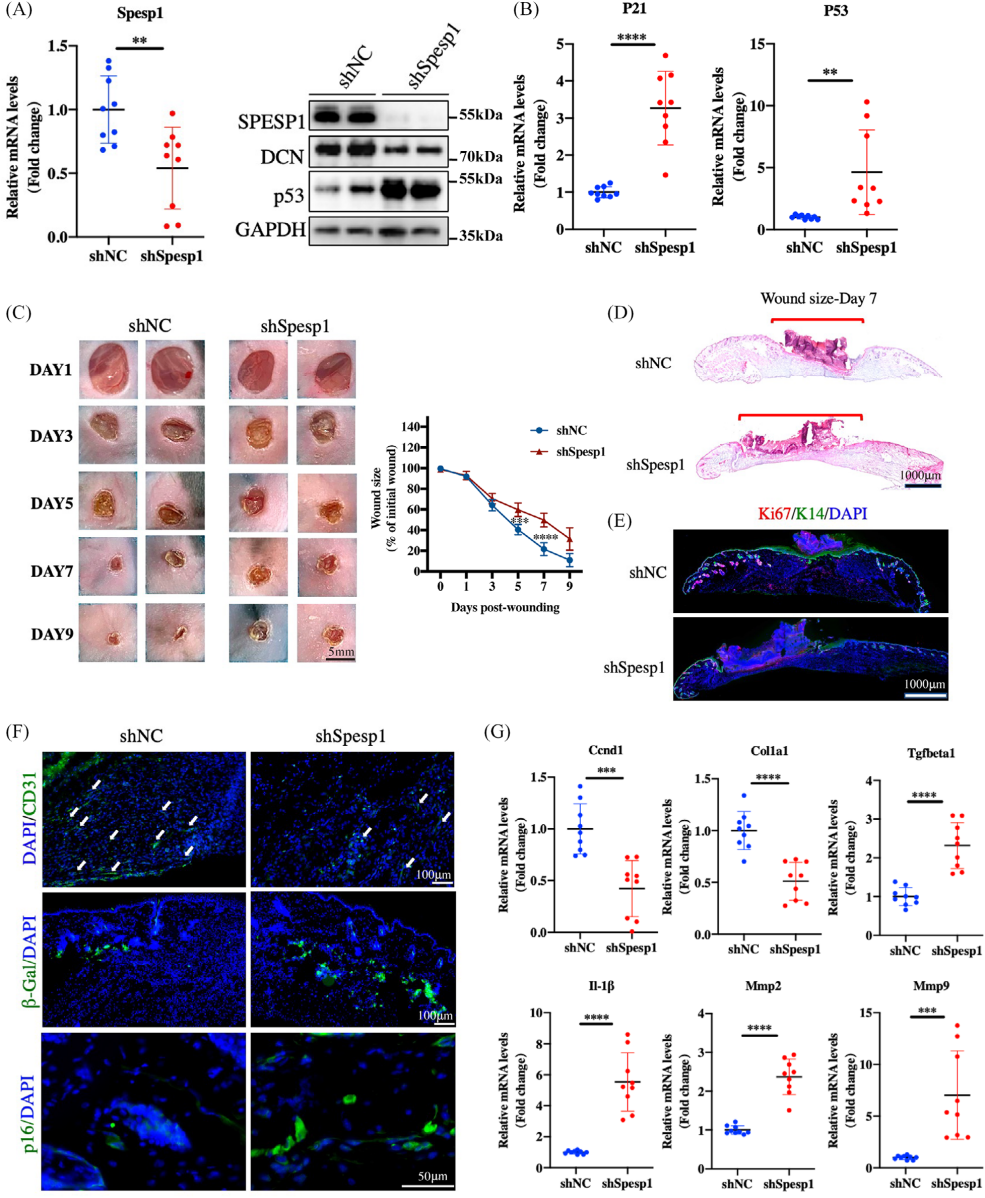
Slowed skin wound healing in Spesp1 knockdown skin. Eight‐week‐old mice were injected with virus fluid and then underwent skin wound modelling surgery. (A) Lentiviruses of shSpesp1 downregulated the mRNA expression of SPESP1 in mouse skin tissue (left). The protein levels of SPESP1, p53 and Dcn were detected by Western blotting (right). (B) Expression of p16 and p53 in mouse skin tissue was measured by qPCR. (C) The skin wound healing process. Representative results of injury after 1 and 9 days are shown, Changes in wound area as the percentage of the initial wound area for 9 days after the injury are shown. (D) Midline sections of the wound with H&E staining 7 days after the injury are shown. (E) Immunofluorescence staining of Ki67 and K14 staining. (F) Immunofluorescence staining of CD31, p16 and β‐Gal staining. (G) The mRNA expression levels of Ccnd1, Col1a1, Il1b, Mmp9, Mmp2 and Tgfbeta1 in shS pesp1 skin. *n* = 6 mice per group, mean age 2 months. The data are shown as the mean ± SEM; ∗*p* < 0.05; ∗∗*p* < 0.01; ∗∗∗*p* < 0.001; ∗∗∗∗*p* < 0.0001; ns, not significant.

Considering SPESP1's influence on the wound healing pathway, we next determined whether SPESP1 is involved in the ageing process and affects wound healing, using lentiviral vector (LV) as a tool to deliver shSpesp1 to the skin. We injected shNC or shSpesp1 lentiviruses subcutaneously into the skin of young mice, and skin samples were collected 5 days post‐injury. The protein and mRNA results showed a successful knockdown of SPESP1 in mouse skin, followed by decreased DCN expression. (Figure 6A). Seven days after injury, the wound size in the shSpesp1 group was notably larger than that in the shNC group (Figure 6C). The skin at the wound site in the shSpesp1 group showed several important ageing features, such as elevated p53, p21 expression and increased β‐Gal and p16 staining (Figure 6B,F and Supplementary Figure 8A). H&E staining revealed that wounds of shSpesp1 mice were more extended than shNC‐treated wounds (Figure 6D and Supplementary Figure 8B). Skin wound healing is a complex and comprehensive process, including re‐epithelialization and the migration, proliferation and differentiation of different skin cells. To judge the re‐epithelialization process and cell proliferation, we collected skin from the injured site of the mice and performed K14 and Ki67 immunostaining. The epithelial tongue of migrating keratin‐forming cells was observed beneath the scab, showing intact migration and enrichment with Ki67+ proliferating cells in shNC skin, but not shSpesp1 skin (Figure 6E and Supplementary Figure 8C). Likewise, the number of CD31‐positive micro‐vessels in shSpesp1 skin was significantly reduced compared with shNC skin (Figure 6F and Supplementary Figure 8D). In addition, the mRNA results showed that the expression of genes related to cell cycle and collagen synthesis (Ccnd1 and Col1a1) was downregulated, while the expression of genes associated with inflammation and collagen degradation (Tgfbeta1, Il1β, Mmp3 and Mmp9) was upregulated in the shSpesp1 group (Figure 6G).

Next, we asked whether SPESP1 overexpressed could accelerate wound healing. Endogenous overexpression of SPESP1 by injection of overexpressing virus fluid protected mouse skin from the senescent phenotype and accelerated wound healing, as evidenced by attenuated senescent phenotype, the extent of wound healing and accelerated angiogenesis (Supplementary Figure 9A–G). Taken together, these findings indicate that SPESP1 expression correlates with delayed skin healing in ageing skin.

### 2.7 Clearance of senescent cells ameliorates SPESP1 knockdown‐delayed wound healing

**FIGURE 7:**
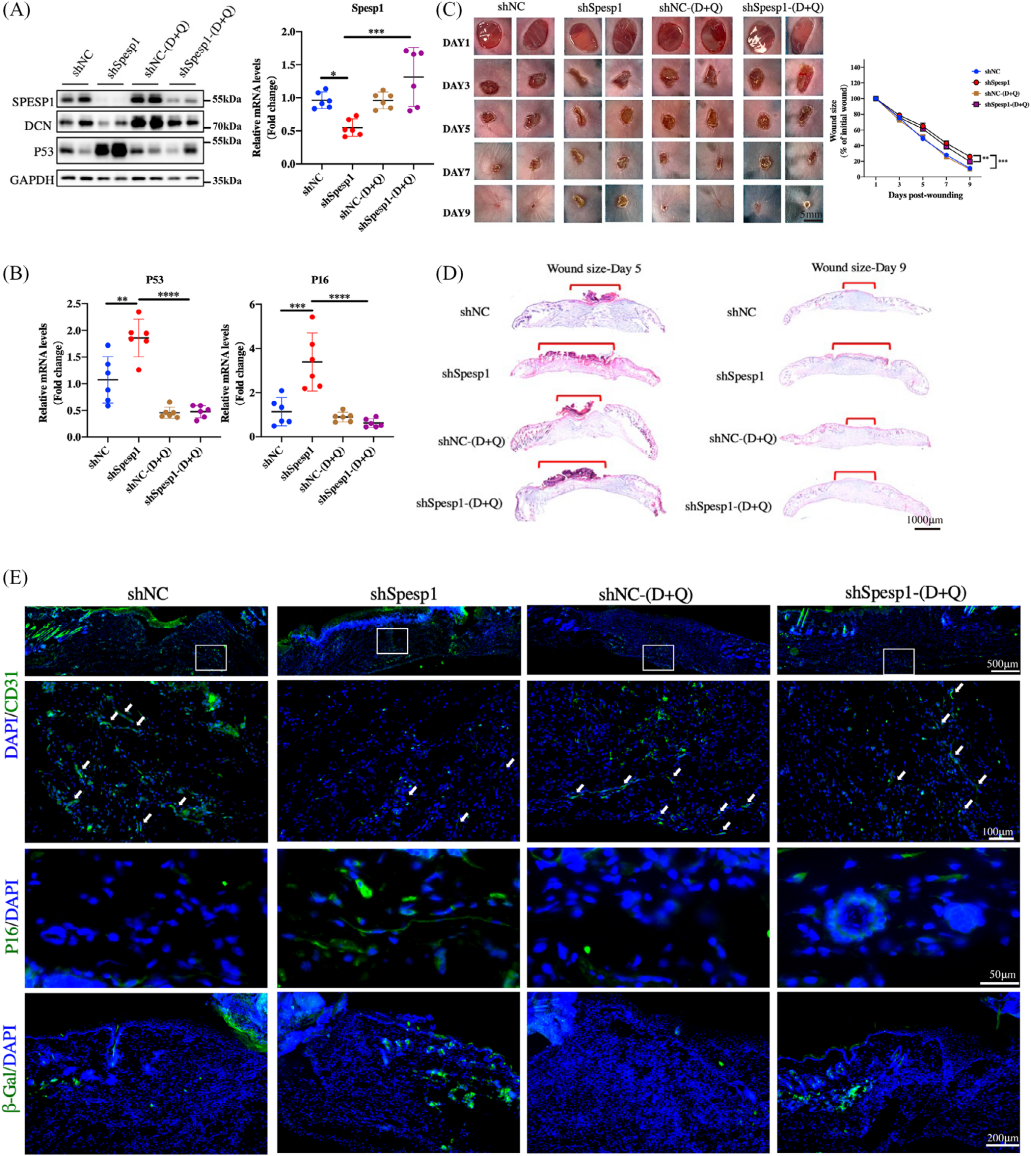
Senolytic drugs improved wound healing in shSpesp1 mice by clearing senescent cells. Eight‐week‐old mice were injected with virus fluid and given 5 mg/kg dasatinib plus 50 mg/kg quercetin or vehicle by gavage, then underwent skin wound modelling surgery. (A) The expression of Spesp1 in wound tissue was determined by qPCR (right) and WB (left). The protein levels of p53 and Dcn by Western blotting. (B) The expression of p16 and p21 in wound tissues was determined by qPCR. (C) The skin wound healing process. Representative results of wound healing from day 1 to day 9, changes in wound area as the percentage of the initial wound area for 9 days after the injury are shown. (D) Midline sections of the wound with H&E staining 7 days after the injury are shown. (E) Immunofluorescence staining of CD31, p16 and β‐Gal staining. *n* = 6 mice per group, mean age 2 months. The data are shown as the mean ± SEM; ∗*p* < 0.05; ∗∗*p* < 0.01; ∗∗∗*p* < 0.001; ∗∗∗∗*p* < 0.0001; ns, not significant.

Previous studies have reported that senescent cells delay healing in old individuals, such as fracture healing.^29^, ^30^ In ageing skin, senescent fibroblast accumulation and a local environment resembling a low‐level but persistent chronic inflammatory state could be the contributors to delayed wound healing.^31^ To determine whether there is a correlation between senescence cells and healing delay induced by SPESP1 knockdown, we repeated the D+Q experiment in shNC and shSpesp1 wounded mice and assessed SPESP1, DCN and senescence markers (p53 and p21) expression. The protein expression of both SPESP1 and DCN was remarkably reduced in the traumatized tissues of shSpesp1 mice, but both were reversed after D+Q treatment (Figure 7A and Supplementary Figure 10A). Moreover, D+Q treatment reduced Spesp1sh‐induced senescence markers p16, p53, p21 and β‐Gal‐positive cells and significantly promoted wound healing, (Figure 7B–E). However, in mice treated with shNC, D+Q had minimal to no impact on ageing markers or wound healing. As shown in Supplementary Figure 10B, D+Q accelerated re‐epithelialization and promoted proliferating cells in the shSpesp1 group, yet no substantial change was observed in the shNC group. Moreover, D+Q treatment also increased angiogenesis and the expression levels of Ccnd1 and Col1a1, while reducing SASP expression in the wound area of the shSPESP1 group (Supplementary Figure 10C). These results indicated that delayed wound healing observed in SPESP1‐knockdown skin is, in part, attributed to the accumulation of senescent HDFs induced by SPESP1 shRNA.

## 3 DISCUSSION

Similar to other tissues, the skin undergoes unpreventable intrinsic ageing.^1^, ^32^ Wound healing is impaired in the elderly, leading to health complications and attracting great attention.^33^, ^34^, ^35^ However, the molecular underpinnings of delayed wound healing in elderly individuals are poorly understood.^10^, ^30^ In this study, we demonstrate the critical role of SPESP1 in inducing senescence in HDFs, subsequent skin ageing and delayed wound healing. This suggests that targeting the SPESP1/MECP2/DCN axis could potentially mitigate the progression of skin ageing and age‐associated delay in healing.

Dermal fibroblasts are the primary senescent cells accumulating in aged skin.^36^ Senescent HDFs not only diminish collagen production, contributing to skin ageing and sagging but also influence the tissue micro‐environment, potentially leading to the development of ageing‐associated degenerative disorders.^37^, ^38^, ^39^ Here, we observed a significant downregulation of SPESP1 in senescent HDFs and ageing skin. Furthermore, SPESP1 knockdown induced cell senescence and accelerated skin ageing. While previous studies have linked SPESP1 to the growth and development of tumour diseases,^20^, ^40^, ^41^, ^42^ there remains a limited understanding of the functional roles and underlying mechanisms of SPESP1.

A key molecular mechanism contributing to skin ageing involves alterations in the extracellular matrix,^43^, ^44^ notably a decline in mature collagen, which is essential for maintaining skin elasticity and tissue strength.^45^, ^46^ Transcriptome analysis indicated that DCN could be the target gene of SPESP1 in skin ageing. DCN, as an extracellular matrix protein, represents the most abundant proteoglycan in the skin,^47^ playing an essential role in protecting collagen from degradation by binding to collagen to stabilize collagen fibres.^48^, ^49^ DCN knockout mice presented thin dermis and fragile skin with irregular collagen fibres.^50^ Moreover, the downregulation of DCN was also observed in aged skin.^51^ Our research adds to this understanding by indicating that DCN acts as a downstream of SPESP1, alleviating the ageing phenotype induced by SPESP1 knockdown in the skin.

Over the past few decades, research has shown that ageing is characterized by a series of hallmark features, one of which involves the alteration of cellular epigenetics.^52^, ^53^ The most meaningful thing at the moment is to extensively explore the epigenetic changes caused by ageing under physiological conditions^54^ and thus to discover the condition‐specific ageing regulation mechanism. MeCP2 is an epigenetic regulator that binds to methylated DNA.^55^ MECP2 inhibits the expression of target genes by binding to methylated DNA via its methylcytosine‐binding domain region.^56^, ^57^ It has been documented that MeCP2 can regulate the transcription of FOXO3a by methylating its promoter region, thus influencing cellular functions.^58^ MeCP2 binds to the promoter of pro‐inflammatory cytokine II‐6 and negatively regulates its expression in ischemia‐reperfusion injury of the kidney.^59^ Here, we found that SPESP1 can bind to the MBD of MeCP2 to reduce hypermethylation of the DCN promoter region. At the same time, more studies believe that MeCP2 only acts as a methylation‐binding protein, the involvement of other methylation‐modifying proteins such as DNA methyltransferases (DNMT) or ten eleven translocation (TET) in the regulation of DCN cannot be excluded.

In wound healing, dermal fibroblasts are the main effector cells^15^ and play an important role in wound contraction, collagen synthesis and tissue remodelling.^60^, ^61^ Moreover, postnatal neointima formation occurs primarily through angiogenesis, which provides oxygen and nutrients to the wound to sustain fibroblast proliferation, collagen synthesis and re‐epithelialization.^15^ In the current study, we observed that the downregulated SPESP1 resulted in a delay in wound healing. Moreover, elevated ageing markers and reduced DCN were observed in the skin with delayed healing. We, therefore, suggest that the effect of SPESP1 on wound healing comes from two aspects. First, DCN secreted by HDFs contributes to delayed healing by diminishing the healing function of senescent HDFs. Second, senescent HDF‐induced ageing surrounding the environment further delays wound healing in the skin. This observation aligns with findings by Liu et al., who noted that cellular senescence hampers fracture healing in the elderly.^30^ Likewise, growing evidence suggests that eliminating senescent cells is an effective way to restore tissue homeostasis and prolong healthy lifespan in mice.^62^, ^63^ In this study, we also found that clearance of senescent cells by D+Q treatment improved wound healing in ageing skin.

There are still some limitations in this study. Although our co‐localization analysis shows that SPESP1 is predominantly expressed in HDFs, fibroblast‐specific knockdown of SPESP1 mice is lacking, which could further clarify the important role of HDFs senescence in skin ageing. Second, the role of DCN in wound healing affected by SPESP1 needs further confirmation.

## 4 CONCLUSIONS

In summary, our study reveals that the interaction between SPESP1 and MeCP2 induces downregulation of DCN expression, consequently inducing HDF senescence and subsequent skin ageing. Moreover, our investigation sheds light on the role of cellular senescence in wound healing, providing compelling evidence for the potential therapeutic targeting of SPESP1 to ameliorate the ageing phenotype.

## 5 METHODS

### 5.1 Animals

Female C57BL/6J mice (6–8 weeks old, 18–22 g) were purchased from SLAC Laboratory Animals Co., Ltd. Aged mice (>18 months old) were obtained from the Genetic Laboratory of Central South University. All these mice were maintained in specific pathogen‐free conditions at this facility. The Animal Ethics Committee of Xiangya Hospital, Central South University approved all research and experimental protocols.

Mice were housed in individually ventilated cages. The day before skin wound modelling (day −1), both young and older mice were anesthetized with an intraperitoneal injection of avertin (40 mg/kg), followed by the removal of fur from their dorsal region. Next (day 0, 1), LVs designed to either overexpress or knock down the SPESP1 gene, along with an LV‐empty vector as a control, were prepared. These LVs were concentrated and then diluted in phosphate‐buffered saline (PBS) provided by Gibco, Thermo Fisher Scientific, USA, to achieve a concentration of 10^6^ (TU) in a 50‐µL volume. We then administered 50 µL of these LVs intradermally at the base and periphery of pre‐marked dorsal skin wound sites in the mice, ensuring uniform distribution of the injections. After two consecutive days of lentivirus injection (day 2), a skin wound was made on the back using a 6 mm biopsy punch (Miltex) to a depth reaching the sub‐dermis of the skin and kept open throughout the healing process. The wound area of the control and lentivirus‐injected mice was observed every post‐wounding day. Mice were euthanized 5, 7 and 9 days post‐injury, and the skin tissue after the injury was collected after death (*n* = 6 animals/per vector/time point). The diameter of each wound taken is approximately 1 cm, approximately 2−3 mm beyond the boundaries of the initial wound including the surrounding uninjured skin.

### 5.2 Human skin tissues

Normal human skin samples were collected from individuals undergoing skin surgery for non‐malignant lesions in the Department of Dermatology, Xiangya Hospital, Central South University. The hospital's Ethics Committee approved this procedure. Participants were categorized into two different groups: the young group, 18–29 years old (*n* = 8; mean age 23.8) and the old group, 60–82 years old (*n* = 6; mean age 76.5). The information on skin samples from individuals of different ages is listed in Appendix Table S2.

### 5.3 Cell culture

HDFs) were isolated from the foreskin of healthy individuals (A: 16 years old; B: 24 years old) undergoing clinical circumcision. These donors had provided informed consent as per the protocols approved by the Clinical Research Ethics Committee of Xiangya Hospital, Central South University. The harvested cells were primarily cultured in Dulbecco's modified Eagle's medium (supplied by Thermo Fisher Scientific, USA), enriched with 10% heat‐inactivated foetal bovine serum (Biological Industries). 293T cells were obtained from the Cyagen Bioscience. All cells were incubated at 37°C in 10% humid air and 5% CO_2_. Passages < 15 were used as young HFDs and passages > 35 were used as old senescent HFDs in this study.^64^

### 5.4 Antibodies

Antibodies used in this study include SPESP1 (H00246777‐B01P, Abnova) (28010‐1‐AP, Proteintech), DCN (14667‐1‐AP, Proteintech), MeCP2 (ab253197, Abcam) (10861‐AP, Proteintech), p16 (ab108349, Abcam), p21 (2947S, Cell Signaling Technology), HSP90 (13171‐1‐AP, Proteintech), CD31 (558736, BD Biosciences), p53 (sc‐126, Santa Cruz), Ki67 (SAB5700770, Sigma), Anti‐Flag (Sigma, catalogue F3165), Anti‐HA (Cell Signal Technology, catalogue 3724S) and GAPDH (ab‐8245, Abcam). Secondary antibodies were purchased from Zsbio company and Abcam.

### 5.5 Cellular senescence model of HDFs

For hydrogen peroxide (H_2_O_2_)‐induced senescence, cells were exposed to H_2_O_2_ (400 µM) for 2 h. Control group cells were simulated treatment (an equal volume of PBS) and cells were collected 3 days later.

For UVA‐induced senescence, after replacing PBS, the cells were placed in an ultraviolet radiometer (Opsytec Dr. Groebel, Germany), continuously irradiated with UVA 10 J/cm^2^ for 3 days, and the cells were collected after 24 h.

### 5.6 Lentiviral plasmid construction

SPESP1/DCN short hairpin RNA and PLVX‐IRES‐SPESP1/DCN lentiviruses encoding full‐length SPESP1/DCN cDNA were developed by Sangon Biotech (Shanghai) Co., Ltd. These were designed to silence and overexpress SPESP1/DCN, respectively. We used a triple plasmid system to transfect HEK293T cells to produce recombinant lentivirus. The target vector (5 µg), Vesicular stomatitis virus G (VSVG) (3.2 µg) and delta R (1.8 µg) were co‐transfected into HEK293T cells. After 2 days, the supernatant was concentrated with PEG8000 and finally resuspended in PBS (100 µL). Stable cell lines were selected using 0.5 µg/mL puromycin starting at 48 h post‐infection for 1 week. Infected cell samples were named shSPESP1/shDCN or SPESP1/DCN. The expression of SPESP1/DCN was verified before further experiments. We injected 50 µL of lentivirus intradermally into the same pre‐labelled site and marked the same size of skin at multiple sites at a dose of 10^6^ TU. We kept the injection evenly distributed to ensure widespread transduction efficiency in the skin at the marked sites. This process was repeated every 2 days for 2 months to maintain consistent expression of target genes (*n* = 5 female C57BL/6J mice /per vector). Details regarding the primer sequences utilized in this study can be found in Appendix Table S1.

### 5.7 Senolytic intervention

To assess the efficacy of D + Q senolytic therapy in mitigating skin ageing induced by shSpesp1, we divided 2‐month‐old female C57BL/6J mice into four distinct groups: (i) shNC group: placebo; (ii) shNC group: D + Q treatment; (iii) shSpesp1 group: placebo; and (iv) shSpesp1 group: D + Q treatment (*n* = 10). 5 mg/kg Dasatinib (S5254; Selleck Chemicals) plus 50 mg/kg quercetin (S2391; Selleck Chemicals) was prepared in 10% polyethylene glycol 400 (Sigma‐Aldrich 91893). D + Q was given orally by gavage in 100 µL, 2 days apart each time (*n* = 6 animals/per group).

While transfecting the virus solution, cells were subjected to a vehicle (0.1% DMSO) or a combination of dasatinib (200 nM) and quercetin (20 µM) until sample collection and then the senescence phenotype of cells was detected.

### 5.8 Real‐time quantitative PCR

Total RNA was extracted from the collected cell or tissue samples using TRIzol, followed by transcription into cDNA using the cDNA Synthesis Kit (K1682, Thermo Fisher Scientific). The quantitative real‐time polymerase chain reaction (qRT‐PCR) was performed using the AceQ Universal SYBR qPCR Master Mix (Vazyme) in a fluorescent quantitative PCR system (Bio‐Rad) with glyceraldehyde‐3‐phosphate dehydrogenase (GAPDH) serving as the normalization reference. The relative expression levels of mRNA were quantified employing the 2‐ΔΔCT method with each experiment conducted at least thrice. Primer sequence details utilized within this study are documented in Appendix Table S1.

### 5.9 Western blot

HDFs after different treatments were collected, rinsed with cold PBS and lysed using RIPA buffer (P0013B, Beyotime). After centrifugation, the protein content was quantified following the BCA Protein Assay protocol (23227, Thermo Fisher Scientific) protocol. Equal concentrations (30 µg) of samples were loaded on sodium dodecyl sulfate‐polyacrylamide gel electrophoresis (SDS‐PAGE) gels and transferred to polyvinylidene difluoride membranes (Millipore, Bedford, MA, USA). Protein on the membrane is blocked at room temperature for 1 h and then washed with Tris‐buffered saline with Tween 20 (TBST). Membranes were incubated with primary antibody and shaken overnight at 4°C. Following this, the membranes were thrice washed with TBST and incubated with heavy chain and light chain (H&L) secondary antibodies (goat anti‐mouse/rabbit IgG, ZSGB‐BIO, ZB‐2301/2305).

### 5.10 SA‐β‐gal staining

The treated HDFs were counted and replated into a 35‐mm Petri dish (6×10^4^ cells). After 24 h, the growth medium was removed, cells were rinsed with PBS and then fixed with 1 mL of β‐Galactosidase Fixation Solution (Beyotime Biotechnology) at room temperature for 15 min. Post‐fixation, cells were washed thrice with PBS. Then, 1 mL of staining working solution was added to each well and incubated overnight at 37°C.

Wound tissue was rapidly frozen in optimal cutting temperature (O.C.T.) compound (Tissue‐Tek) and sectioned continuously (6 µm thickness) using a Leica CM1860 cryostat. Frozen tissue sections were stained using the CellEvent Senescence Green Detection Kit (C10851, Thermo Fisher Scientific).

### 5.11 Cell apoptosis assay

HDFs were collected and suspended with 110 µL binding buffer. The cells were then stained with FITC‐An‐nexin V Apoptosis Detection Kit (BD Biosciences), Annexin V Alexa Fluor 488 and propidium iodide and incubated for 30 min at room temperature in the dark. Post‐incubation, an additional 400 µL of binding buffer was added and the cells were subjected to analysis via Cytek Athena flow cytometry.

### 5.12 Cell cycle analysis

The treated fibroblasts were allocated in a six‐well plate (5×10^4^ cells/per well), and the cells and supernatant were collected and centrifuged. Following this, we employed the Cell Cycle and Apoptosis Analysis Kit (Beyotime, C1052) to stain the cells. This step was conducted in a dark environment at ambient temperature for 30 min. After incubation, cells were washed and analysed using a flow cytometer.

### 5.13 5‐ethynyl‐2'‐deoxyuridine (EdU)  staining

The treated fibroblasts were placed on a 96‐well plate (3×10^3^ cells/per well). The cell‐Light EdU Apollo488 In Vitro Kit (RIBOBIO) was utilized for the staining process, adhering to the provided guidelines. The image was taken by the Leica DM5000B microscope.

### 5.14 HE staining

Frozen tissue sections were used for HE staining, first fixed with 4% Paraformaldehyde (PFA) for 10 min, followed by a 10‐min haematoxylin (HE) application, and then briefly differentiated in 1% hydrochloric acid ethanol for 10 s. After rinsing with running water for 10 min eosin staining was applied for 4 min. The sections were then sequentially dehydrated using alcohol concentrations of 80%, 90% and 100%, soaked in xylene twice for 2 min each time and treated with neutral gum to seal the film. Finally, the films were observed under a microscope and photographed.

### 5.15 Immunofluorescence

For immunofluorescence assays, skin sections were freeze‐embedded. Next, wound tissue sections were fixed in 4% PFA for 10 min, washed in PBS three times and blocked for 1 h using 5% donkey serum in PBS. The primary antibody was applied and left to incubate at 4°C overnight. Following this, the slides were washed in PBS and incubated with the corresponding secondary antibody at room temperature for 1 h, then washed again in PBS. Nuclei were stained using DAPI at 1 µg/mL concentration. A Nikon DS‐Ri2 microscope was employed for capturing fluorescence images.

Cells were cultured in 24‐well plates. 1×10^4^ cells were cultured per well. After adherence, cells were washed thrice with PBS and fixed with 4% paraformaldehyde for 10 min at room temperature. Blocking was performed with 5% donkey serum for 1 h. Add primary antibody to cells and incubate overnight at 4°C. The binding of the primary antibody was subsequently detected with a secondary antibody. Staining was observed through fluorescence microscopy.

### 5.16 Sample statistical quantitative information

For each sample, a total of three sections were used for quantitation to ensure a representative analysis of the tissue. These sections of the tissues include the epidermis and dermis from both the wound area and the area nearby the wound. The selected standardized sections include both intact non‐wound tissue and wound section tissue. Images for quantitation were taken at 0, 3, 5, 7 and 9 post‐wounding to capture the dynamic process of wound healing. For wound healing‐related indicators, such as He staining, Ki67 and K14, full‐layer images were taken. For p16 and β‐gal, five images are taken at pre‐determined positions for each slice to ensure full coverage of the tissue area. The area of tissue imaged was determined based on the wound centre and included both the wound edge and adjacent healthy tissue to assess the healing gradient. Image quantitation was performed using ImageJ. For the quantification of stained cultured cells, ImageJ was used to count the number of positive cells or compare the fluorescence intensity in five randomly selected high‐power fields.

### 5.17 RNA sequencing

The total RNA of shNC, shSPESP1 #1 and shSPESP1 #2 cells was extracted with TRIzol reagent (Thermo Fisher Scientific Company). RNA‐Seq was performed at OEBiotech. Co., Ltd (Shanghai). Reads were analysed with Salmon software to calibrate and quantify transcript expression. Genomic comparisons were obtained for each sample by comparing the reads to a reference genome. The counts of genes in each sample were normalized, and differential fold changes were calculated using DESeq2, and differential significance testing was performed using the negative binomial distribution. Finally, genes that were differentially expressed (DEGs) were identified based on the criteria of an absolute log2 fold change (|log2FC|) greater than 1 and an adjusted *p*‐value less than 0.05. GO analysis was performed for the enrichment of DEGs using R packages (‘clusterProfiler’, ‘GOplot’, ‘enrichplot’ and ‘ggplot2’).

### 5.18 Gene silencing with siRNA

Invitrogen Lipofectamine RNAiMAX (Thermo Fisher) was used to transfect untargeted negative control siRNA or MeCP2 siRNA (300 nM) into fibroblasts according to manufacturer's instructions. After 24−48 h of transfection, the cell lysate was collected to extract protein and RNA for further analysis.

### 5.19 Cell counting kit‐8 test

To evaluate the proliferation rate of HDFs, HDFs were placed in 96‐well (3×10^3^ cells per well). 10 µL of CCK‐8 (Sigma‐Aldrich) solution was added to each well on days 0, 1, 2, 3 and 4 after seeding. Samples were then incubated for 2 h. Following a 2‐h incubation period, the absorbance at 450 nm (A450) was measured utilizing a microplate reader (Thermo Fisher, USA). These procedures were independently repeated a minimum of three times to ensure reliability, and averages were generated using measurements from five replicated wells.

### 5.20 Methylation‐specific PCR

Cells were collected from the shNC + NC group, shSPESP1 + NC group, shNC + siMeCP2 group and shSPESP1 + siMeCP2 group for MSP detection. First, cell DNA fragments (TreliefTM Animal Genomic DNA Kit, TsingKe) were extracted, and DNA samples were converted into bisulfite using a DNA bisulfite conversion kit (DP215‐02, TIANGEN). Further purification was performed using the methylation‐specific PCR kit (EM101‐01, TIANGEN). PCR products were analysed with 3% agarose gel and quantified with ImageJ software.

### 5.21 Co‐immunoprecipitation and mass spectrometry

The cell extract was prepared using IP lysis buffer (NP‐40) with protease inhibitors. Take 10% of the supernatant for monitoring protein input, the rest of the sample is first pre‐cleared with magnetic beads for 30 min and then incubated overnight on a rotating wheel at 4°C with flag‐coupled magnetic beads (Bimake). After washing three times with lysis buffer for 5 min, immune complex beads were boiled in 6× Protein Loading Buffer (TransGen Biotech) and then loaded with 10% SDS‐PAGE gel.

For identifying proteins that interact with SPESP1 through mass spectrometry (MS), SPESP1 was overexpressed in HDFs. The lysates from these cells were immunoprecipitated using an anti‐SPESP1 antibody. The resulting eluents were analysed through LC‐MS/MS sequencing and processed by Oebiotech Co., Ltd, Shanghai, China, for data analysis.

### 5.22 Chromatin immunoprecipitation

The ChIP assay was performed using Pierce Sepharose ChIP kit (26156, Thermo Fisher Scientific) for methylation‐level verification. First, the treated fibroblasts were fixed with 1% formaldehyde for 10 min. Glycine was added to quench the fixation. The cells were lysed using micrococcal nuclease (ChIP grade), and the antibody was mixed with pre‐washed protein a‐sepharose beads and incubated at 4°C for 1 h on a rotator. Then 25 µg of sheared chromatin was added to the mixture and incubated on a Ferris wheel shaker at 4°C overnight. As a result, 10% sheared chromatin was obtained as input. Immunoprecipitated chromatin was eluted from beads by spinning with elution buffer at room temperature for 30 min. Immunoprecipitated chromatin and imported chromatin were incubated at 65°C for 4 h to delink chromatin, followed by purification of DNA. Finally, the resulting sample was subjected to qRT‐PCR.

### 5.23 LC/MS‐MS mass spectrometry‐based proteomics

Twenty‐five milligrams of the skin tissue was ground in 200 µL of lysis solution, and the protein was obtained by centrifuging at 12 000×*g* for 20 min. After quantification of protein concentration by bicinchoninic acid assay (Thermo Fisher Scientific, USA), 30 µg protein samples were processed according to the kit instructions (Omicsolution, China). The peptides were then separated by a Thermo NanoVipe‐C18 column (25 cm × 75 mm) using a Vanquish Neo HPLC system (Thermo Fisher Scientific, USA) and analysed using an Orbitrap Exploris 480 mass spectrometer (Thermo Fisher Scientific, USA). Data‐independent acquisition (DIA) raw data was analysed using Spectronaut (v 18).

### 5.24 Proteomics analysis

The limma R package was used for differential expression analysis between the control and Spesp1sh groups. The GO and Kyoto encyclopedia of genes and genomes (KEGG) enrichment of DEPs were performed using the clusterProfiler R package The GSVA analysis was then applied to assess pathway activity, using R packages ‘GSVA’ and ‘GSEABase’. Heatmaps illustrating the various pathways and DEPs were generated using the R package ‘pheatmap.'.

### 5.25 Datasets and the correlation analysis

Proteomic data from primary skin fibroblasts of 82 healthy individuals, aged 22−89 years, were obtained from the research of Dimitrios Tsitsipatis.^65^ The correlation of the protein levels between DEGs and p16 was analysed using the Pearson correlation analysis.

### 5.26 Statistical analysis

All data were analysed using PRISM 8.4.0 software (GraphPad, CA, USA). Results are expressed as mean ± standard deviation (SD). We analysed the normal distribution and similar variances between groups. Student's *t*‐test was applied for two‐group comparisons, while one‐way analysis of variance (ANOVA) or multi‐factor ANOVA was used for multiple group comparisons. For nonnormally distributed data or when variances were unequal, the two‐tailed Mann–Whitney U test was employed. A *p*‐value < 0.05 was considered indicative of statistical significance.

## AUTHOR CONTRIBUTIONS

Yun Zhong conducted the experiments, analysed the data, and wrote the draft; Lei Zhou, Yi Guo, Fanping He, Yufan Cheng, Fang Wan, Xin Meng and Hongfu Xie provided technical support and research resources; Yiya Zhang and Ji Li designed the study, arranged the data and wrote the manuscript. All authors have discussed and approved the final version of the manuscript.

## CONFLICT OF INTEREST STATEMENT

The authors have declared that no competing interest exists.

## ETHICS APPROVAL AND CONSENT TO PARTICIPATE

All the study was approved by Xiangya Hospital of Central South University.

## CONSENT FOR PUBLICATION

The authors consented to publish the manuscript.

## Supporting information

Supporting Information

## Data Availability

The data and the code that support the findings of this study are available at reasonable request from the corresponding authors.
