# Supplementary material for: Downregulated SPESP1‐driven fibroblast senescence decreases wound healing in aged mice
Source: Clin Transl Med. 2024 May 19;14(5):e1660. doi: 10.1002/ctm2.1660 (PMC11103130; doi:10.1002/ctm2.1660)
Supplement: Supplementary file 1 — Supporting Information [file CTM2-14-e1660-s001.docx]

Downregulated SPESP1-driven fibroblast senescence decreases wound healing in aged mice

Yun Zhong^1,2^, Lei Zhou^2,3^, Yi Guo^1,2^, Fan Wang^1,2^, Fanping He^1,2^, Yufan Cheng^1,2^, Xin Meng^1,2^, Hongfu Xie^1,2^, Yiya Zhang^1,2,4 *^, Ji Li^1,2,4 *^

1. Department of Dermatology, Xiangya Hospital, Central South University, Changsha, P.R. China.

2. Hunan key laboratory of aging biology, Xiangya Hospital, Central South University, Changsha, P.R. China.

3. Department of Dermatology, the Third Affiliated Hospital, Sun Yat-sen University, Guangzhou, P.R. China.

4. National Clinical Research Center for Geriatric Disorders, Xiangya Hospital, Central South University, Changsha, Hunan, P.R. China, 410008.

*** Corresponding author.**

Ji Li: liji_xy@csu.edu.cn, Tel: +86 731 84327472;

Yiya Zhang: [yiya0108@csu.edu.cn](mailto:yiya0108@csu.edu.cn)

# Supplemental figure


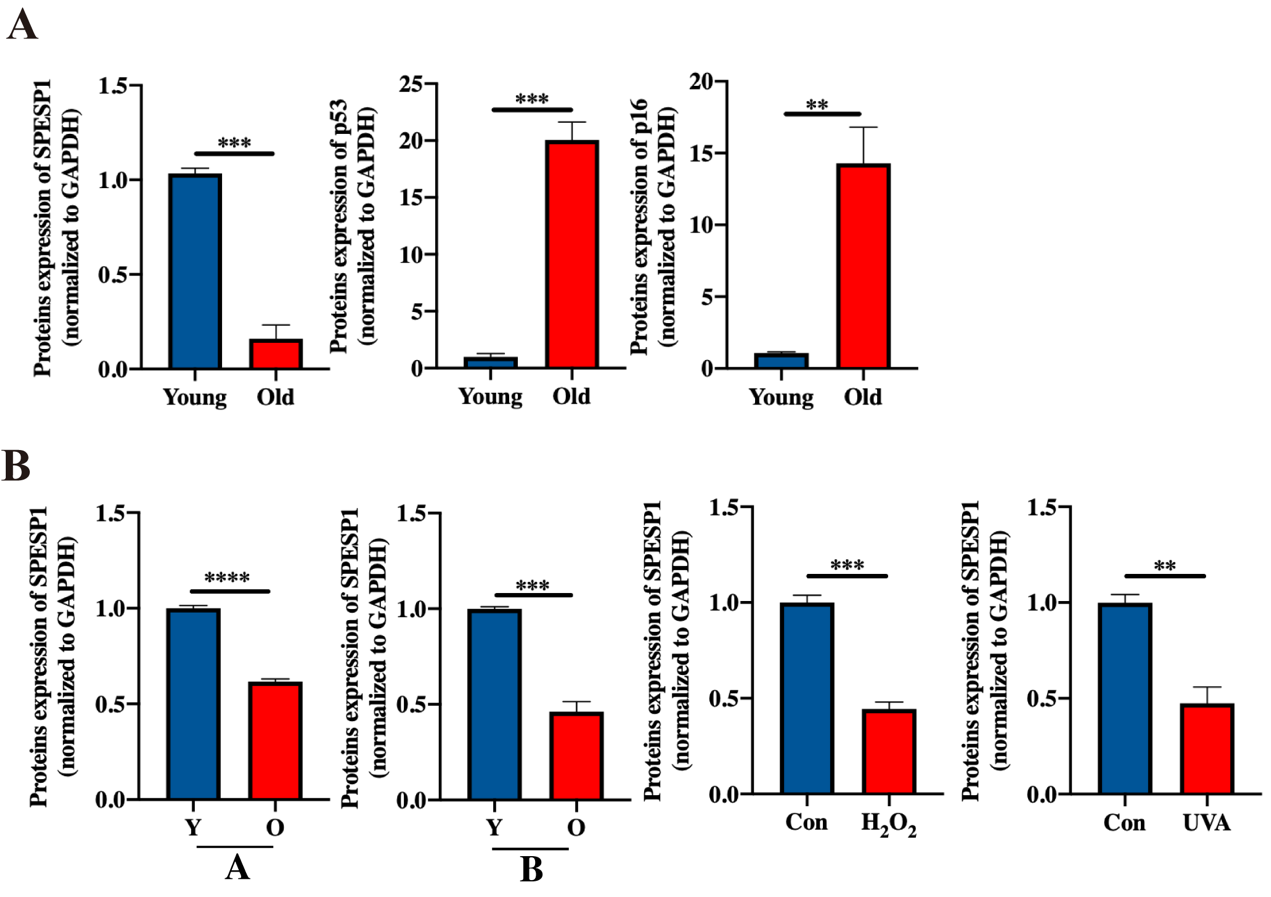


## Figure. S1 SPESP1 delayed HDFs senescence and skin aging.

(A) Protein expression of SPESP1, p53 and p16 in the skin from Young (n=8, mean age 2 month) and Old (n=8, mean age 20 month) mice. (B) Protein expression of SPESP1 in passage senescence, UVA-induced senescence, and H_2_O_2_-induced senescence. Data are shown as mean ± SEM. *P < 0.05; **P < 0.01; ***P < 0.001.


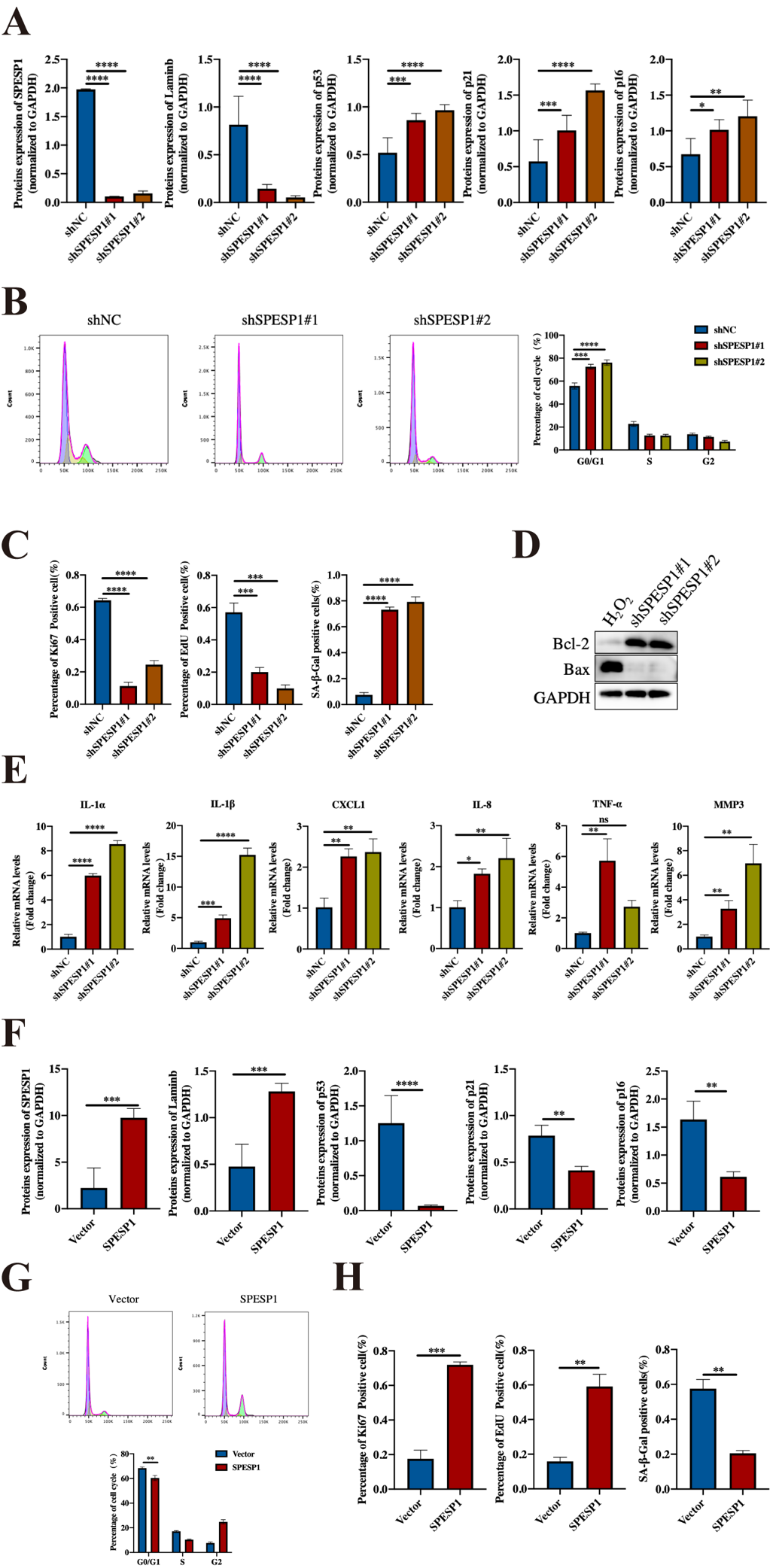


## Figure. S2 SPESP1 delayed HDFs senescence and skin aging.

(A) Protein expression of SPESP1, Laminb, p53, p21 and p16 in shNC and shSPESP1 HDFs. (B) Cell cycle analysis of SPESP1 silenced HDFs by flow cytometry. (C) Quantification of ki67、Edu and SA-β-Gal positive cells in shNC and shSPESP1 HDF. (D) The proteins levels of BCL-2 and Bax after SPESP1 knockdown by western blotting. (E) The mRNA expression levels of SASP after knockdown of SPESP1. (F) Protein expression of SPESP1, Laminb, p53, p21 and p16 in Vector and SPESP1 overexpressed HDFs. (G) Cell cycle analysis of overexpressed SPESP1 HDFs by flow cytometry. (H) Quantification of ki67、Edu and SA-β-Gal positive cells in Vector and SPESP1 overexpressed HDF. Data are shown as mean ± SEM. *P < 0.05; **P < 0.01; ***P < 0.001.


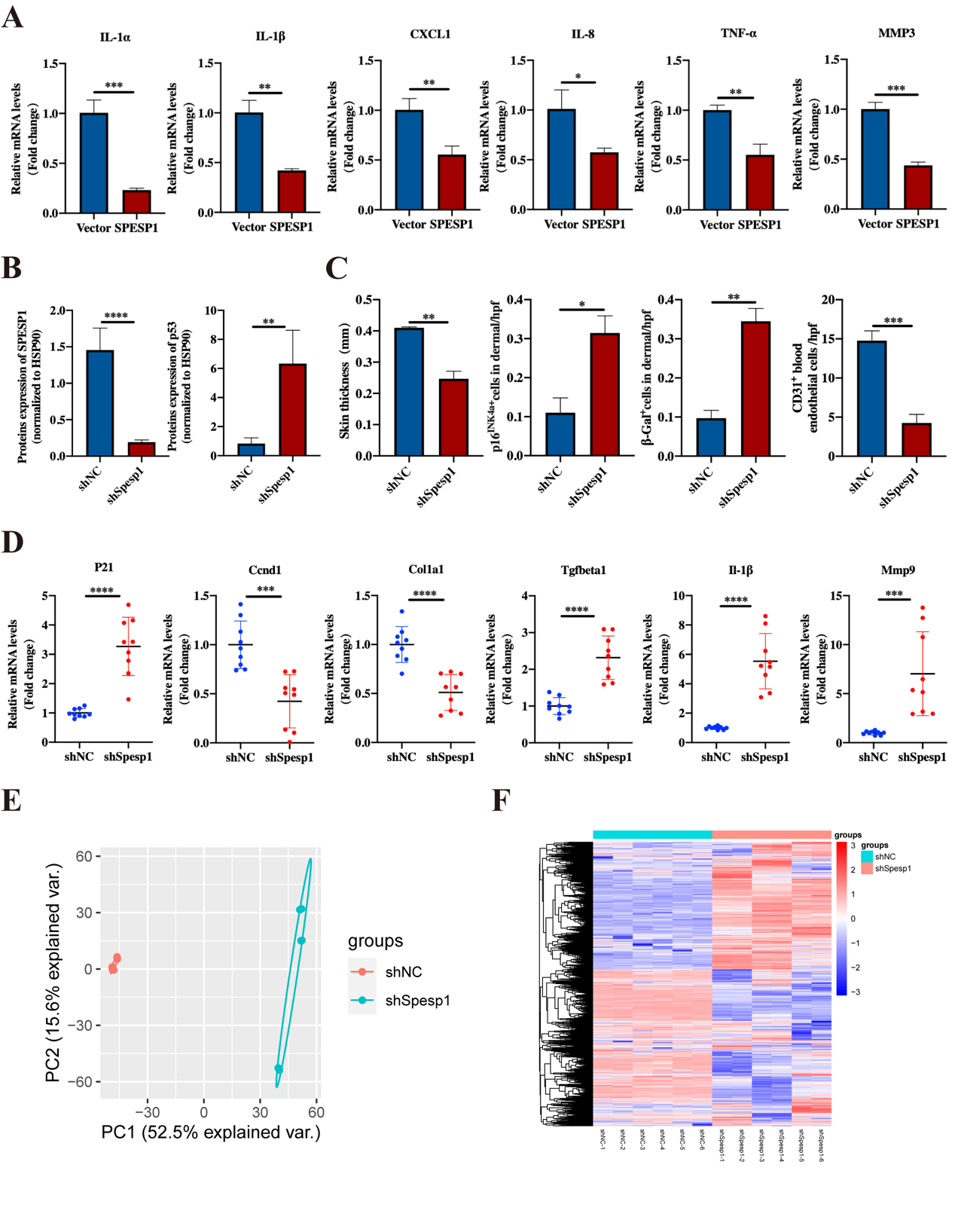


## Figure. S3 SPESP1 delayed HDFs senescence and skin aging.

(A) The mRNA expression levels of SASP after SPESP1 overexpression . (B) Protein expression of SPESP1 and p53 in skin with shNC and shSPESP1. (C) Quantification of skin thickness and p16 ^INK4a+^、β-Gal^+^、CD31^+^blood endothelial cells in skin with shNC and shSPESP1. n=9 skins from 5 mice per group, mean age 2 month. (D) The mRNA expression levels of p21, Ccnd1, Col1a1, Il1b, Mmp9 and Tgfbeta1 in mice skin with shNC and shSPESP1. (E) The PCA analysis of skin proteome. (F) The heatmap of DEPs. n=9 skins from 5 mice per group, mean age 2 month. Data are shown as mean ± SEM. *P < 0.05; **P < 0.01; ***P < 0.001.


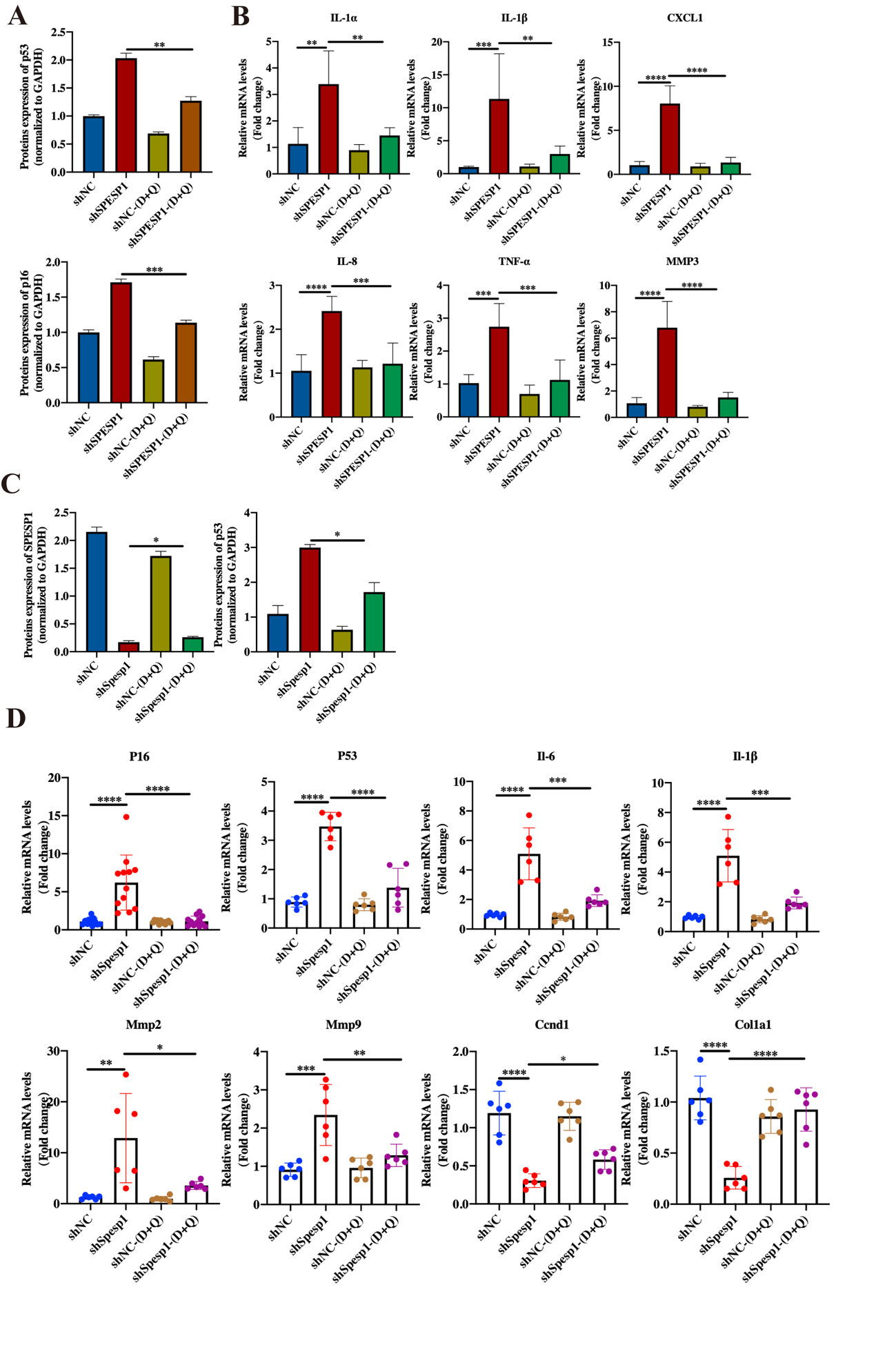


## Figure. S4 Senolytic drug delays skin aging in shSPESP1 knockdown mice by removing SCs

(A) Protein expression of p16 and p53 in D+Q-treated shSPESP1 HDFs. (B) The expression of SASP was measured by qRT-PCR after D+Q treatment in HDF with shNC and shSPESP1. (C) Protein expression of SPESP1 and p53 in D+Q-treated mice skin with shNC and shSPESP1. (D) The expression of SASP was measured by qRT-PCR in D+Q-treated mice skin with shNC and shSPESP1. n=6 mice per group, mean age 2 month. Data are shown as mean ± SEM. *P < 0.05; **P < 0.01; ***P < 0.001.


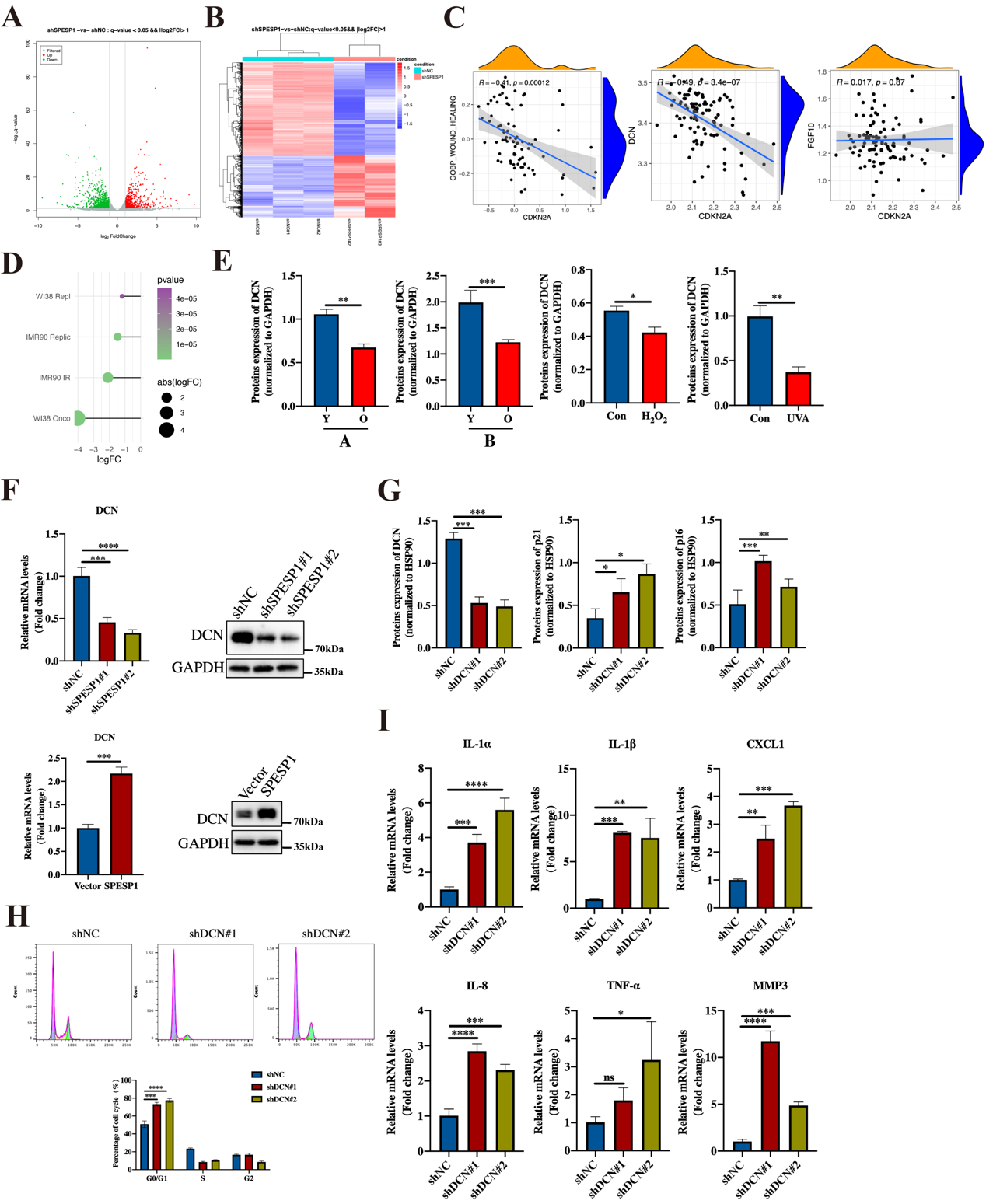


## Figure. S5 RNA-seq and experimental verification revealed DCN as a downstream gene regulated by SPESP1.

(A, B) The volcano map and heatmap of DEGs. High or low expression is indicated by red or green in the digram. (C) Correlation analysis between wound healing pathway activity and CDKN2A(p16) expression; Correlation analysis between DCN/FGF10 and CDKN2A(p16) protein levels. (D) The DCN expression in IR/Onco/Replic-induced senescent IMR90 (Human embryo lung fibroblasts) and WI38 (human embryonic lung fibroblasts) in GSE130727 dataset. (E) Protein expression of DCN in HDFs of passage senescence, UVA-induced senescence, and H_2_O_2_-induced senescence. (F) Expression of DCN was examined by RT-qPCR and western blotting in HDFs with SPESP1 knockdown or overexpression . (G) Protein expression of DCN, p53 and p21 in HDFs with shNC and shDCN. (H) Cell cycle analysis of HDFs with DCN knockdown by flow cytometry. (I) The expression of SASP was measured by qRT-PCR in HDF treated with shNC and shDCN. Data are shown as mean ± SEM. *P < 0.05; **P < 0.01; ***P < 0.001.


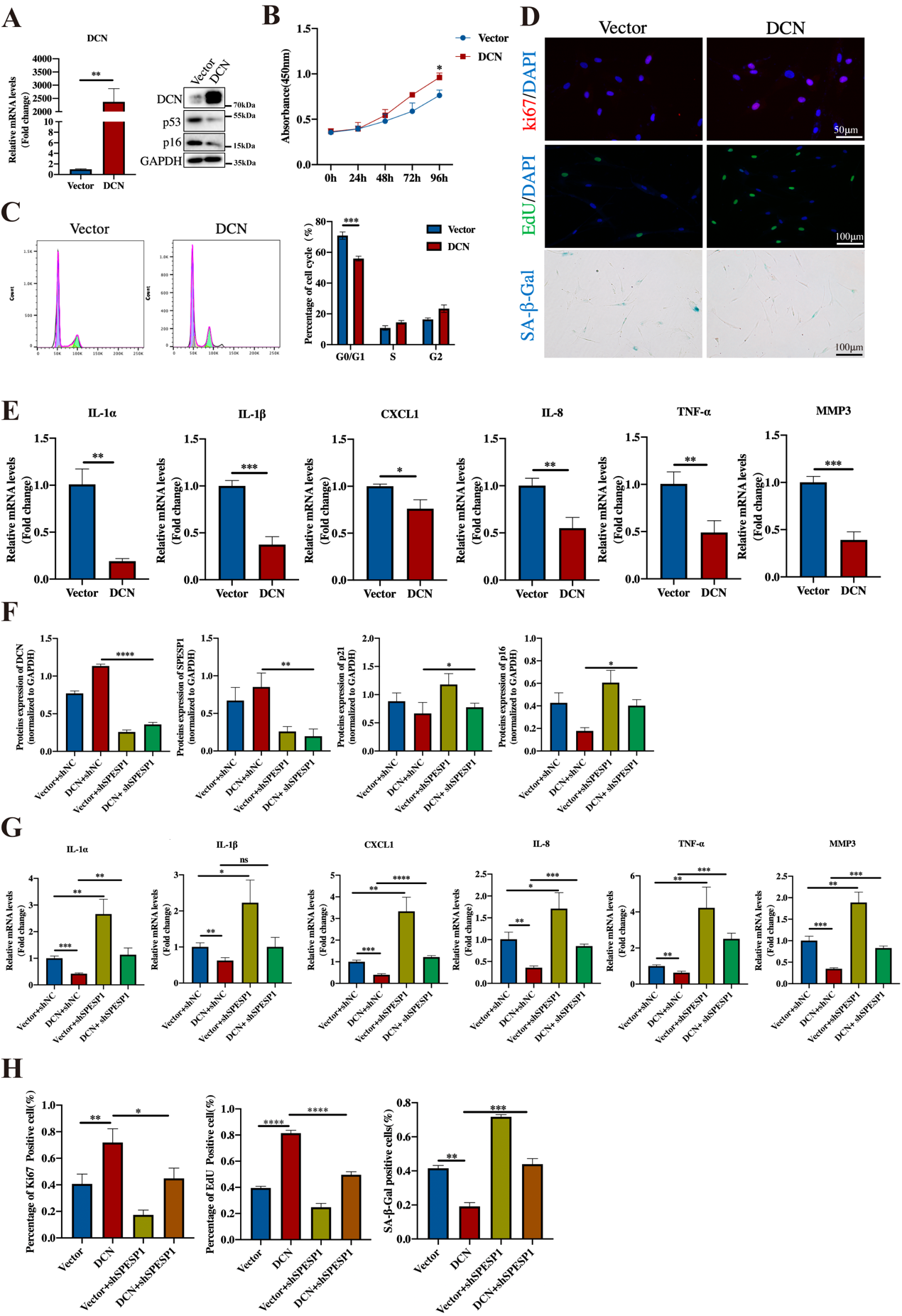


## Figure. S6 DCN was involved in SPESP1-mediated cellular senescence in HDF.

(A) The mRNA expression of DCN (Left), the proteins levels of DCN, p53 and p16 by western blotting (Right) in HDFs with DCN lentivirus. (B) Cell proliferation measured by CCK8. (C) Cell cycle analysis of HDFs with overexpressed DCN by flow cytometry. (D) Immunofluorescence staining of Ki67, Edu, and SA-β-Gal staining in HDFs with overexpressed DCN. (E) The expression of SASP was measured by qRT-PCR in HDF treated with Vector and DCN. Rescue assay was used to assess the reversed effect of DCN in shSPESP1-induced senescence. (F) Protein expression of DCN, SPESP1, p21 and p16 in Vector, DCN, Vector + shSPESP1 and DCN + shSPESP1 treated HDFs. (G) The expression of SASP was measured by qRT-PCR in Vector, DCN, Vector + shSPESP1 and DCN + shSPESP1 treated HDF. (H) Quantification of ki67, Edu and SA-β-Gal positive cells in Vector, DCN, Vector + shSPESP1 and DCN + shSPESP1 treated HDF. Data are shown as mean ± SEM. *P < 0.05; **P < 0.01; ***P < 0.001.


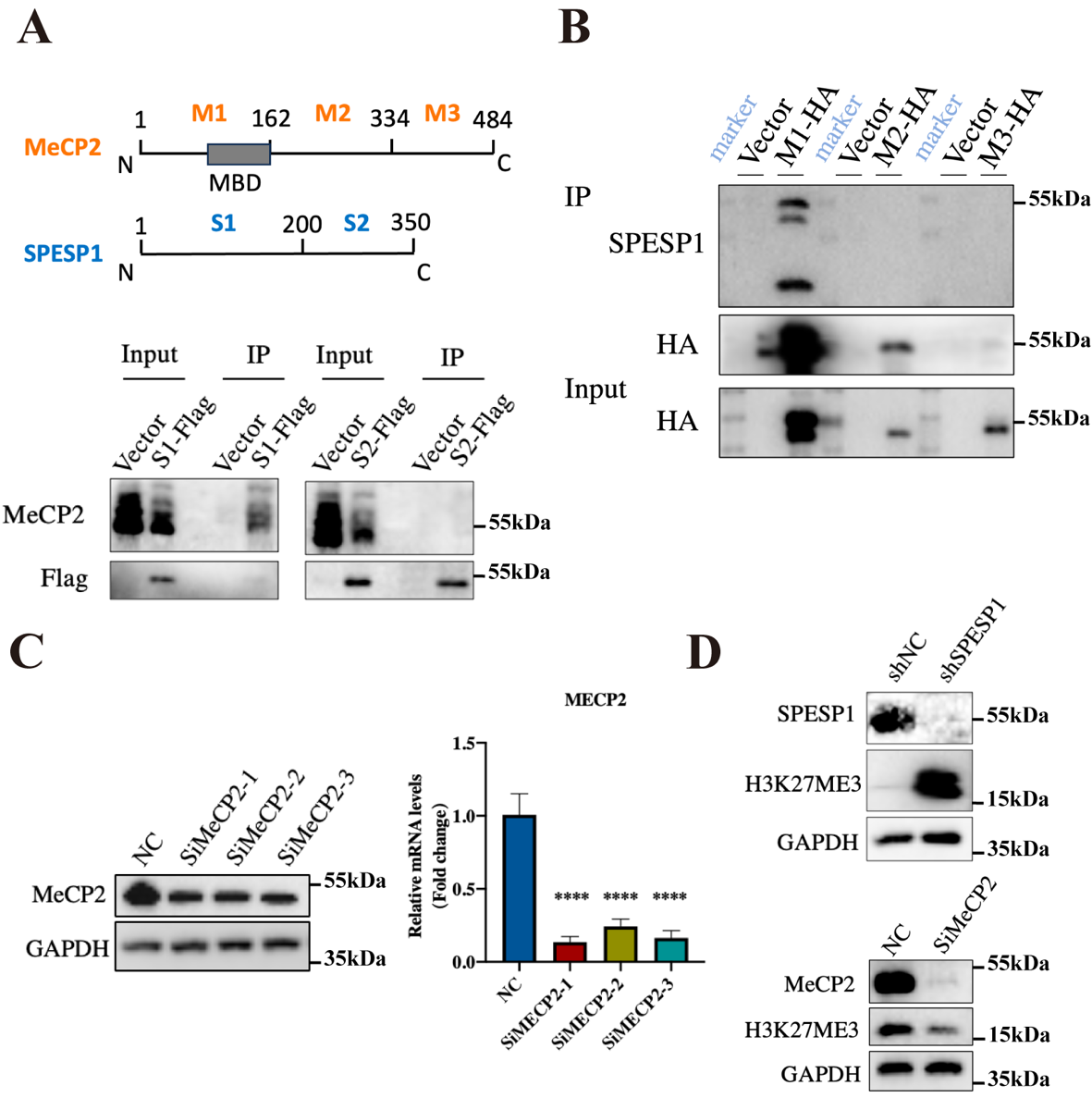


## Figure. S7 SPESP1 binds to MeCP2 to methylate the promoter region of DCN and repress its expression.

(A) (B) The protein structure of MeCP2 was truncated into three regions, namely M1, M2, and M3. M1 contained the methylated DNA binding domain (MBD); the protein structure of SPESP1 was truncated into two regions, namely S1 and S2. The specific binding sites of SPESP1 and MeCP2 were then determined by exogenous co-immunoprecipitation. (C) The knockdown efficiency of siMeCP2 was confirmed by western blot and qRT-PCR. (D) The effect of SPESP1 and MeCP2 knockdown on H3K27ME3 . Data are shown as mean ± SEM. *P < 0.05; **P < 0.01; ***P < 0.001.


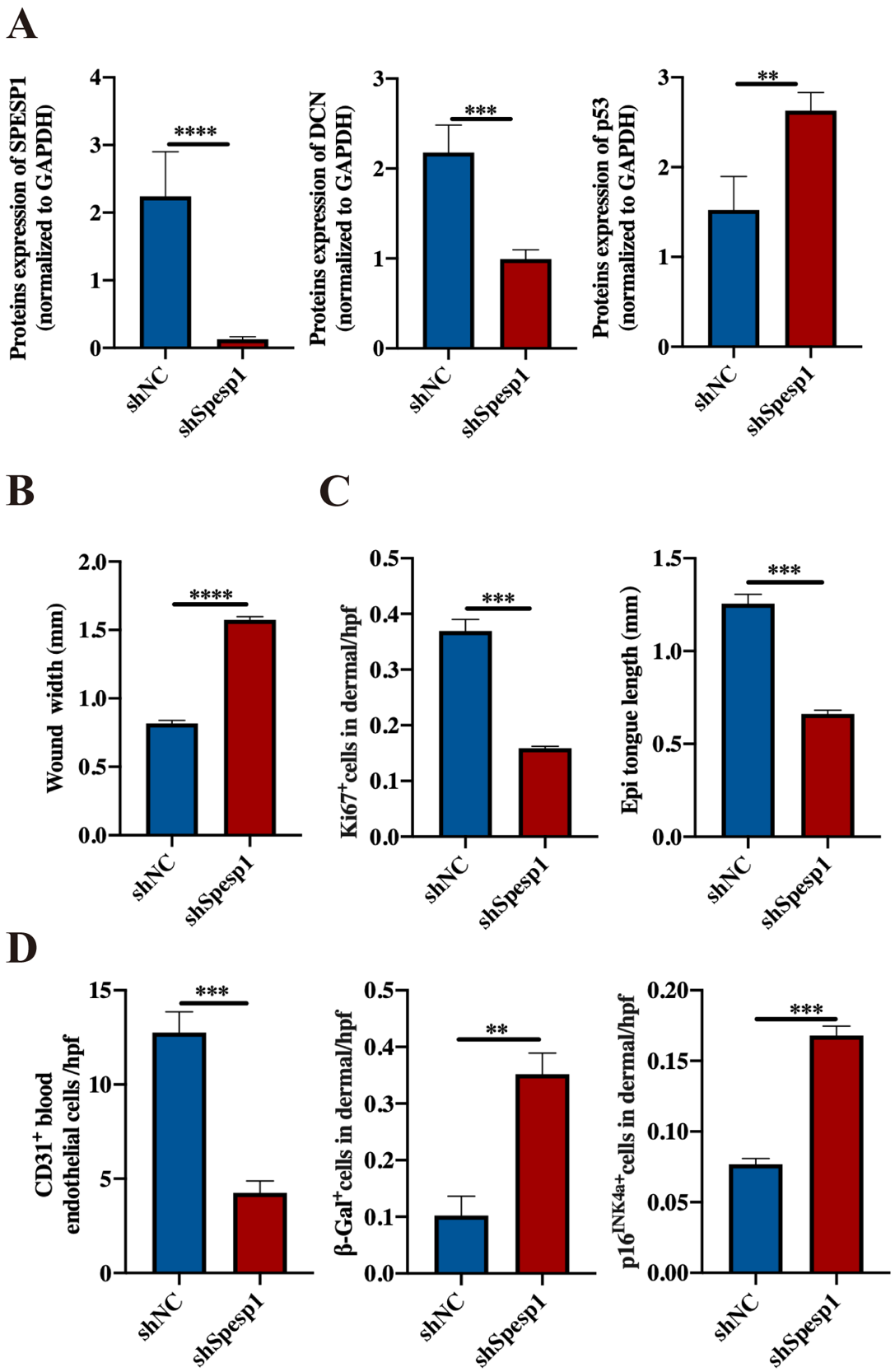


**Figure. S8 Quantitative analysis of shSPESP1 skin wound healing indicators**

(A) Protein expression of DCN, SPESP1 and p53 in skin wounds with shNC and shSPESP1. (B) Quantification of Wound width in skin wounds with shNC and shSPESP1. (C) Quantification of Ki67 positive cells and the length of the tongue of epidermal keratinocytes. (D) Quantification of p16 ^INK4a+^、β-Gal^+^、CD31^+^blood endothelial cells in skin wounds with shNC and shSPESP1. n=6 mice per group, mean age 20 month. Data are shown as mean ± SEM. *P < 0.05; **P < 0.01; ***P < 0.001.


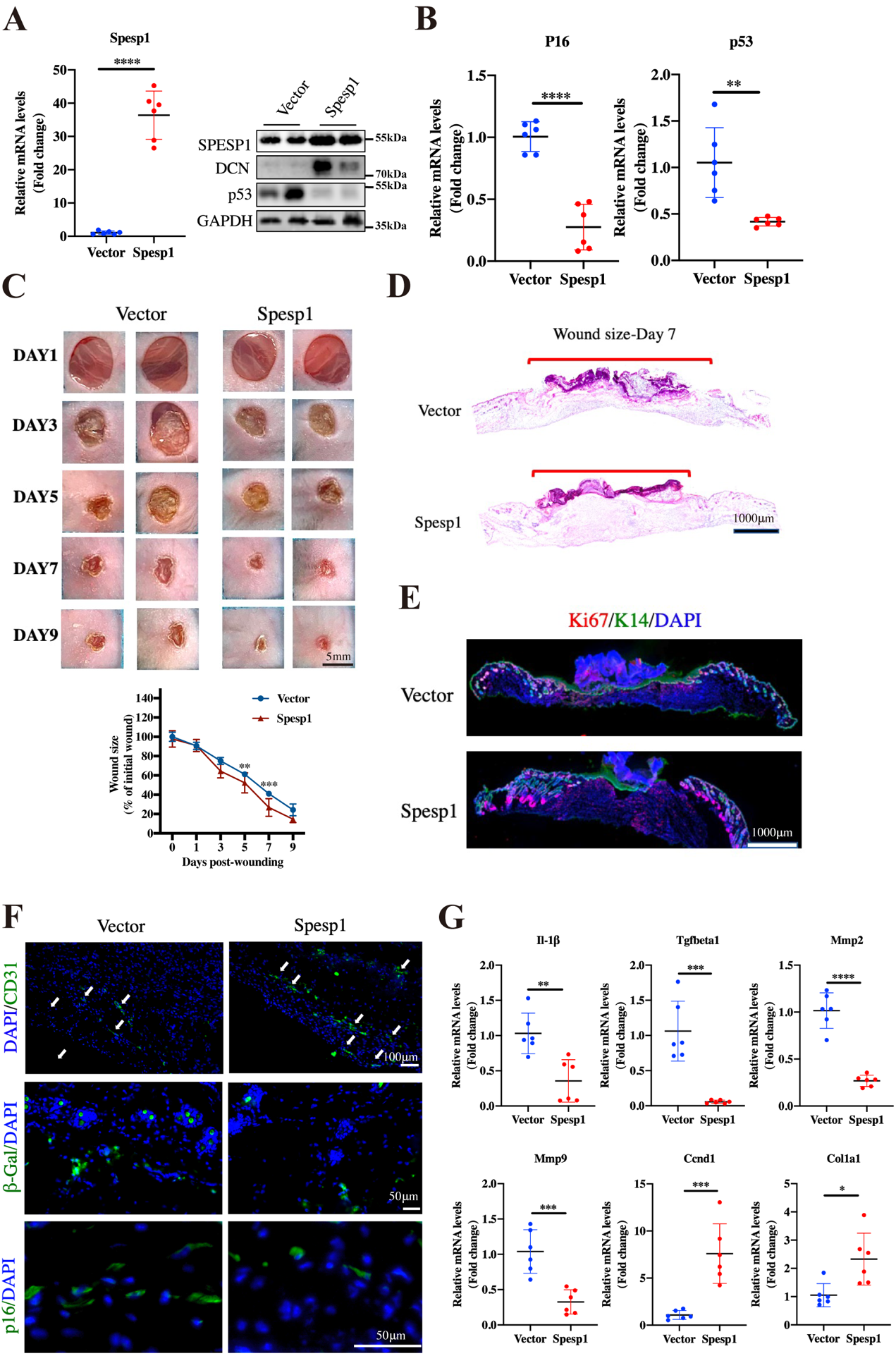


## Figure. S9 Accelerated skin wound healing in Spesp1 overexpressed skin.

20-week-old mice were injected with virus fluid and then underwent skin wound modeling surgery. (A) The mRNA expression of Spesp1, and the proteins levels of Spesp1, p53 and Dcn in mice skin with SPESP1 lentiviruses. (B) The mRNA levels of p16 and p53 in mouse skin tissues. (C) The skin wound healing process is depicted with representative results of injuries observed on days 1 and 9. Changes in the wound area are presented as a percentage of the initial wound area. (D) Midline sections of the wound stained with H&E, at 7 days after the injury. (E) Immunofluorescence staining of Ki67 and K14 staining. (F) Immunofluorescence staining of CD31, p16 and β-Gal. (G) The mRNA expression levels of Ccnd1, Col1a1, Il1b, Mmp9, Mmp2 and Tgfbeta1 after SPESP1 overexpression. n=6 mice per group, mean age 20 month. Data are shown as mean ± SEM. *P < 0.05; **P < 0.01; ***P < 0.001.


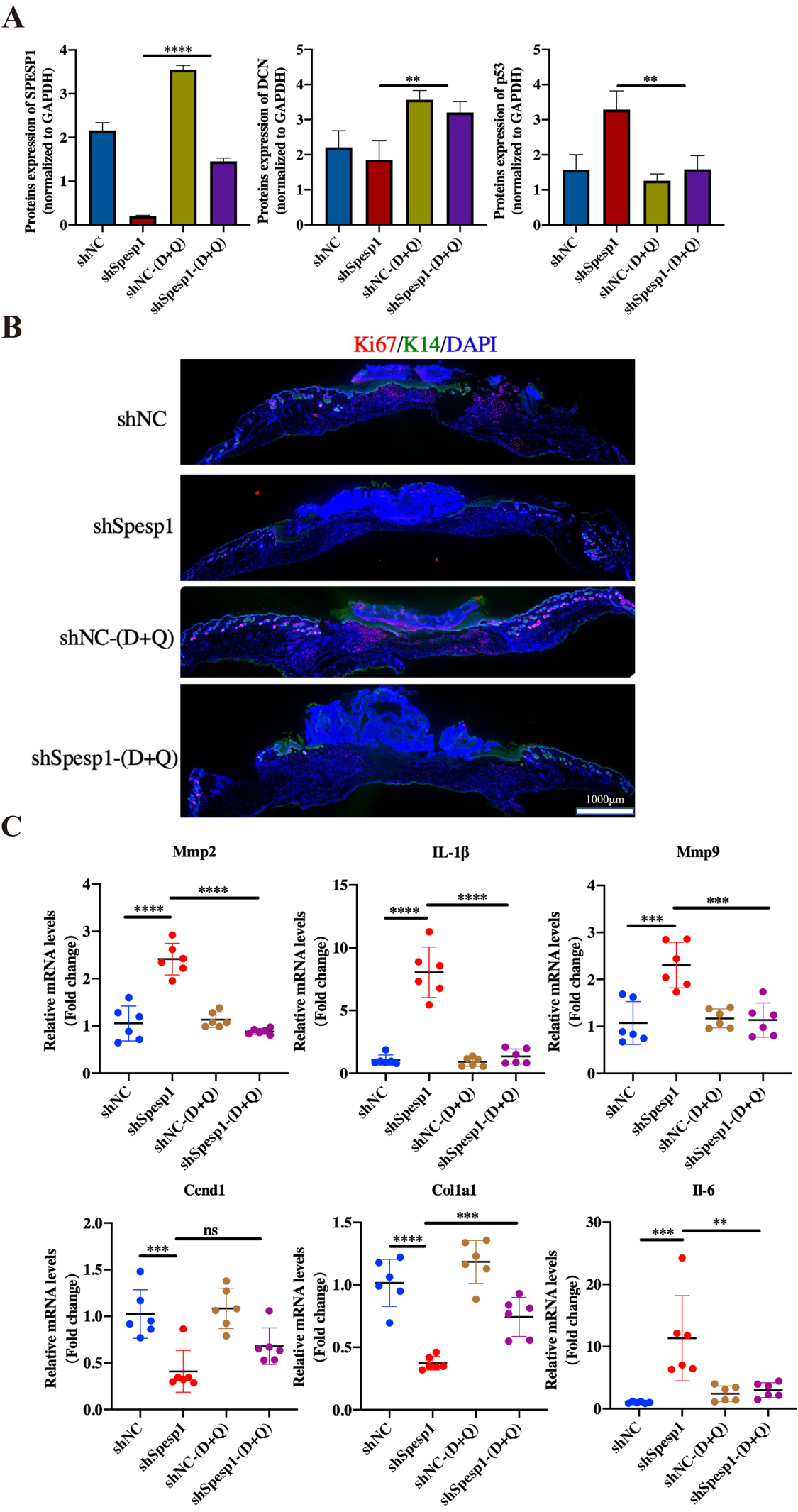


## Figure. S10 Senolytic drugs enhance wound healing in shSPESP1 skin by clearing senescent HDFs.

8-week-old mice were administered virus fluid by injection and subsequently received a combination of 5 mg/kg dasatinib plus 50 mg/kg quercetin by gavage. Following this treatment, the mice underwent skin wound modeling surgery. (A) Protein expression of DCN, SPESP1 and p53. (B) Immunofluorescence staining of Ki67 and K14. (C) The mRNA levels of Ccnd1, Col1a1, Il1b, Mmp9, Mmp2 and Tgfbeta1. n=6 mice per group, mean age 2 month. Data are shown as mean ± SEM. *P < 0.05; **P < 0.01; ***P < 0.001.

## Appendix Table S1. List of primer sequences in this study.

| Gene symbol | Forward primer | Reverse primer |
| --- | --- | --- |
| **Primers for RT-qPCR:** | | |
| Human-GAPDH | GGAGCGAGATCCCTCCAAAAT | GGCTGTTGTCATACTTCTCATGG |
| Human-SPESP1 | GGCTTATCCGAGCATAACTGTG | AGGGAACACTTCGTACTAGGTT |
| Human-DCN | ATGAAGGCCACTATCATCCTCC | GTCGCGGTCATCAGGAACTT |
| Human-MECP2 | GTGGAGTTGATTGCGTACTTCG | CCCTCTCCCAGTTACCGTGAA |
| Human-IL-1β | ATGATGGCTTATTACAGTGGCAA | GTCGGAGATTCGTAGCTGGA |
| Human-IL-1α | ACTCACCTCTTCAGAACGAATTG | CCATCTTTGGAAGGTTCAGGTTG |
| Human-IL-8 | TTTTGCCAAGGAGTGCTAAAGA | AACCCTCTGCACCCAGTTTTC |
| Human-CXCL1 | CATCGAAAAGATGCTGAACAGT | ATAAGGGCAGGGCCTCCT |
| Human-MMP3 | AGTCTTCCAATCCTACTGTTGCT | TCCCCGTCACCTCCAATCC |
| Human-TNF-α | CCTCTCTCTAATCAGCCCTCTG | GAGGACCTGGGAGTAGATGAG |
| Mouse-Gapdh | AGGTCGGTGTGAACGGATTTG | TGTAGACCATGTAGTTGAGGTCA |
| Mouse-Spesp1 | CCTAGTTGCGCTGTGGCTAT | TTTTCCCGAGATCCTGCTCCT |
| Mouse-Il1β | GCAACTGTTCCTGAACTCAACT | ATCTTTTGGGGTCCGTCAACT |
| Mouse-Ccnd1 | AGGCGGATGAGAACAAGCAG | CCTTGTTTAGCCAGAGGCCG |
| Mouse-Col1a1 | TTCTCCTGGCAAAGACGGACTCAA | AGGAAGCTGAAGTCATAACCGCCA |
| Mouse-p21 | CCGAAAACGGAGGCAGACC | CCGAAGATGGGGAAGAGGC |
| Mouse-Mmp9 | CTGGACAGCCAGACACTAAAG | CTCGCGGCAAGTCTTCAGAG |
| Mouse-Tgfb | CTCCCGTGGCTTCTAGTGC | GCCTTAGTTTGGACAGGATCTG |
| Mouse-p16 | CGCAGGTTCTTGGTCACTGT | TGTTCACGAAAGCCAGAGCG |
| Mouse-p53 | ATGAACCGCCGACCTATCC | GGCAGGCACAAACACGAAC |
| DCN promoter | CGAGCTCGGTTTTGAAATATGTCAAGACATAA | CCGCTCGAGCGGAGCCCAAGTAAAAGAGTT |
| **Primer for shRNA:** | | |
| shNC | CCGGCAACAAGATGAAGAGCACCAACTCGAGTTGGTGCTCTTCATCTTGTTGTTTTTG | AATTCAAAAACAACAAGATGAAGAGCACCAACTCGAGTTGGTGCTCTTCATCTTGTTG |
| shSPESP1#1 | CCGGCCAGTTGTTACTGAATCATCTCTCGAGAGATGATTCAGTAACAACTGGTTTTTTG | AATTCAAAAAACCAGTTGTTACTGAATCATCTCTCGAGAGATGATTCAGTAACAACTGG |
| shSPESP1#2 | CCGGCCAGAGAGTTGGAATAATGATCTCGAGATCATTATTCCAACTCTCTGGTTTTTTG | AATTCAAAAAACCAGAGAGTTGGAATAATGATCTCGAGATCATTATTCCAACTCTCTGG |
| shSpesp1 | CCGGTCTGGTCCATCCGGCCAAACAACTCGAGAGACCAGGTAGGCCGGTTTGTT TTTTTTG | AATTCAAAAAATCTGGTCCATCCGGCCAAACAACTCGAGAGACCAGGTAGGCCGGTTTGTT |
| shDCN#1 | CCGGGCCATTCAACTCGGAAACTATCTCGAGATAGTTTCCGAGTTGAATGGCTTTTTG | AATTCAAAAAGCCATTCAACTCGGAAACTATCTCGAGATAGTTTCCGAGTTGAATGGC |
| shDCN#2 | CCGGCCAGGTTGTCTACCTTCATAACTCGAGTTATGAAGGTAGACAACCTGGTTTTTG | AATTCAAAAACCAGGTTGTCTACCTTCATAACTCGAGTTATGAAGGTAGACAACCTGG |
| PLVX-SPESP1 | CGGAATTCCGATGAAGCCCTTAGTCCTT | CCCTCGAGGGATAAACTTTTAATAAGGCTGTGACTCT |
| PLVX-DCN | CGGAATTCATGAAGGCCACTATCATCCT | CGGGATCCTTACTTATAGTTTCCGAGTTGAATGG |

## Appendix Table S2. Information on skin samples from individuals of different ages.

| Donor ID | Age | Sex | Harvest Site | Sun Exposure | Reason for tissue harvest | Medications |
| --- | --- | --- | --- | --- | --- | --- |
| 1 | 19 | F | Upper limbs | Yes | Excision of superficial skin tumors | None |
| 2 | 22 | M | Abdomen | No | Excision of superficial skin tumors | None |
| 3 | 22 | M | Upper limbs | Yes | Excision of superficial skin tumors | None |
| 4 | 23 | M | Upper limbs | Yes | Excision of superficial skin tumors | None |
| 5 | 24 | F | Back | No | Excision of superficial skin tumors | None |
| 6 | 26 | M | Back | No | Excision of superficial skin tumors | None |
| 7 | 26 | M | Back | No | Excision of superficial skin tumors | None |
| 8 | 28 | F | Back | No | Excision of superficial skin tumors | None |
| 9 | 71 | M | Back | No | Excision of superficial skin tumors | None |
| 10 | 75 | M | Back | No | Excision of superficial skin tumors | None |
| 11 | 76 | M | Abdomen | No | Excision of superficial skin tumors | None |
| 12 | 78 | F | Chest | No | Excision of superficial skin tumors | None |
| 13 | 78 | F | Upper limbs | Yes | Excision of superficial skin tumors | None |
| 14 | 81 | M | Back | No | Excision of superficial skin tumors | None |
